# Revealing the Chemical and Structural Complexity of Electrochemical Ion Exchange in Layered Oxide Materials

**DOI:** 10.1021/jacs.4c08089

**Published:** 2024-09-17

**Authors:** Linqin Mu, Dong Hou, Emily E. Foley, Minyi Dai, Jin Zhang, Zhisen Jiang, Muhammad Mominur Rahman, Yanbao Fu, Lu Ma, Enyuan Hu, Sami Sainio, Dennis Nordlund, Jue Liu, Jia-Mian Hu, Yijin Liu, Raphaële J. Clément, Feng Lin

**Affiliations:** † Department of Chemistry, Virginia Tech, Blacksburg, Virginia 24061, United States; ‡ School for Engineering of Matter, Transport and Energy, Arizona State University, Tempe, Arizona 85287, United States; ∥ Institute for Materials Research and Innovation (IMRI), University of Louisiana at Lafayette, Lafayette, Louisiana 70503, United States; ⊥ Materials Department and Materials Research Laboratory, University of California Santa Barbara, Santa Barbara, California 93106, United States; # Department of Materials Science and Engineering, University of Wisconsin-Madison, Madison, Wisconsin 53706, United States; ∇ Stanford Synchrotron Radiation Lightsource, SLAC National Accelerator Laboratory, Menlo Park, California 94025, United States; ○ Energy Storage and Distributed Resources Division, Lawrence Berkeley National Laboratory, Berkeley, California 94720, United States; ◆ National Synchrotron Light Source II, Brookhaven National Laboratory, Upton, New York 11973, United States; ¶ Chemistry Division, Brookhaven National Laboratory, Upton, New York 11973, United States; △ Neutron Scattering Division, Oak Ridge National Laboratory, Oak Ridge, Tennessee 37831, United States; ▲ Department of Materials Science and Engineering, Virginia Tech, Blacksburg, Virginia 24061, United States

## Abstract

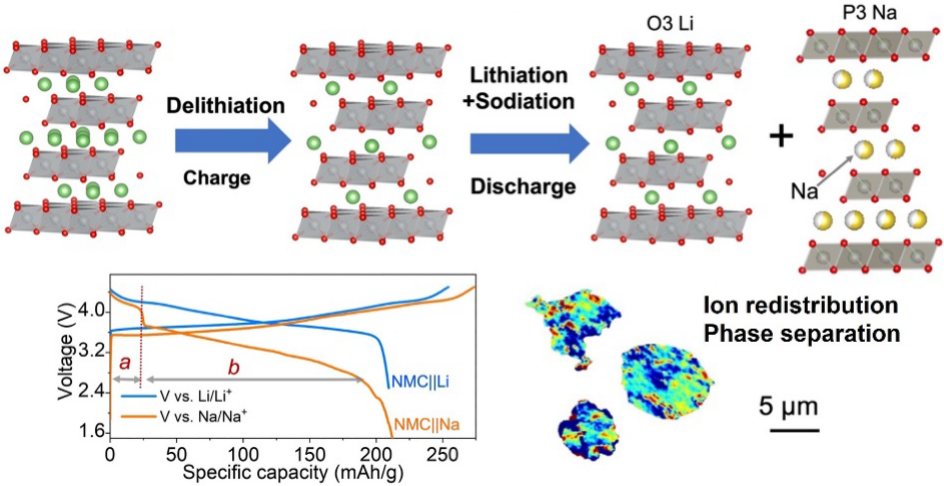

Soft chemistry techniques, such as ion exchange, hold great potential for the development of battery electrode materials that cannot be stabilized via conventional equilibrium synthesis methods. Nevertheless, the intricate mechanisms governing ion exchange remain elusive. Herein, we investigate the evolution of the long-range and local structure, as well as the ion (de)intercalation mechanism during electrochemical Li-to-Na ion exchange initiated from an O3-type lithium-layered oxide cathode. The *in situ*-formed mixed-cation electrolyte leads to competitive intercalation of Li and Na ions. While Li ion intercalation predominates at the beginning of initial discharge, Na ion cointercalation into a different layer results in ion redistribution and phase separation, with the emergence of a P3–Na phase alongside an O3–Li phase. Further, this study spatially resolves the heterogeneous nature of electrochemical ion exchange reactions within individual particles and provides insights into the correlations between local Ni redox processes and phase separation. Overall, electrochemical ion exchange leads to a mixed-phase cathode and alters its reaction kinetics. Those findings have important implications for the development of new metastable materials for renewable energy devices and ion separation applications.

## Introduction

Ion intercalation is a versatile process that involves ion extraction and reinsertion, accompanied by redox processes and structural changes in the bulk of a material. Transition metal oxides with a layered structure have received significant attention as secondary battery electrodes thanks to their ability to accommodate a wide range of mobile cations with varying radii and charges (e.g., H^+^, Li^+^, Na^+^, K^+^, Zn^2+^, Ca^2+^).^1−3^ Numerous transition metal oxides (*A_x_TM*O_2_, *A* is the mobile cation, and *TM* is the transition metal) with varying layer-stacking sequences have been developed by modulating both the *A* and *TM* chemistry,^1,3−6^ making this materials class highly versatile with tunable intercalation properties.^1^ Furthermore, the incorporation of a mixture of alkali metal cations into the interlayer space of layered oxides has been shown to enhance their structural stability and battery performance. For instance, introducing Li into Na-layered cathodes,^7^ Na into Li-layered cathodes,^8^ or K into Na-layered cathodes,^9^ can mitigate layer gliding processes and delay phase transitions during ion (de)intercalation, thus enhancing their structural stability.

The ability to accommodate various mobile ions within a layered structure also opens opportunities for synthesizing novel metastable materials and for examining the links between electro-chemo-mechanics and phase behavior. Thermodynamically stable compounds are usually obtained using equilibrium synthesis methods, e.g., high-temperature solid-state reactions. In contrast, cation exchange through (electro)chemical methods is an effective approach to synthesize metastable compounds with new crystal structures, chemical compositions, and a tunable intercalation chemistry.^10,11^ By utilizing Li^+^/Na^+^ or Na^+^/K^+^ exchange reactions, researchers have successfully obtained unique structures that are otherwise impossible to synthesize through traditional methods, thereby allowing for the modulation of their properties as battery electrode materials.^12−17^ For example, O2-type Li-excess oxide cathodes, derived from the P2-type Na analogues, exhibit a significantly higher capacity and enhanced resistance to irreversible TM migration compared to their O3-type conterparts.^18^ Furthermore, cation exchange also offers a new platform for investigating fundamental questions regarding electrode materials, such as the possibility of phase separation under local nonequilibrium conditions.^17,19−21^ These nonequilibrium conditions can be created by local strains induced by a heterogeneous distribution of ions and redox states across domains. For example, polycrystalline cathode materials, including LiNi_1–*x*–_*_y_*Mn_*x*_Co_*y*_O_2_, often display grain-to-grain phase heterogeneity, which can be further explored through ion exchange.^22,23^

As ion exchange has become a popular strategy to design new materials, a mechanistic understanding of this process is timely. Meanwhile, the study of electrochemical ion exchange can offer insights into the evolution of the ion insertion and extraction properties and phase stability of important electrode materials during battery operation. It is understood that the evolution of the long-range and local structures, including the distribution of cations in the interlayer space, depends on the relative fraction and radii of the exchanging ions. Specifically, a large mismatch between the ionic radii of the exchanging species can complicate the process, especially when a larger cation replaces a smaller cation, usually leading to severe structural distortions and in some cases phase separation.^17,20,24,25^ For example, Ceder and coworkers used K^+^ to replace Na^+^ in Na_*x*_Ni_2_SbO_6_ during electrochemical cycling and found that ion exchange was hindered by Na^+^ redistribution and phase segregation.^17^ Yet, several fundamental questions remain to be answered. The spatial distribution of exchanged ions within the electrode structure remains unclear, particularly as a function of the state of charge. Second, it is unclear how ion exchange evolves as a function of cycling and, at the material level, how it impacts the (de)intercalation properties over time. At the cell level, the presence of mixed alkali metal ions and their continuous exchange during electrochemical cycling likely also impact cell performance, including but not limited to changes to the voltage profile, capacity, and rate capability, although those effects have been overlooked.

Herein, we study the evolution of the long-range and local structure and the ion (de)intercalation properties of the Ni-rich LiNi_0.8_Mn_0.1_Co_0.1_O_2_ cathode during electrochemical Li/Na ion exchange. We investigate the phase evolution of the material using a variety of *operando* and *ex situ* characterizations, including diffraction, spectroscopy, and imaging techniques. The *in situ*-formed mixed-cation electrolyte results in competitive intercalation of Li and Na ions. Generally, upon discharge, Li intercalation takes place prior to (at higher potentials than) Na intercalation; further, Na intercalation leads to a redistribution of the Li ions and phase separation within a single particle. Specifically, a P3-type Na-dominating phase grows alongside an O3-type Li-dominating phase. Overall, the ion exchange process results in long-range and local structural changes during cycling that are substantially more complex than for a single ion intercalating cathode.

## Results and Discussion

### Electrochemical Ion Exchange

**Figure 1:**
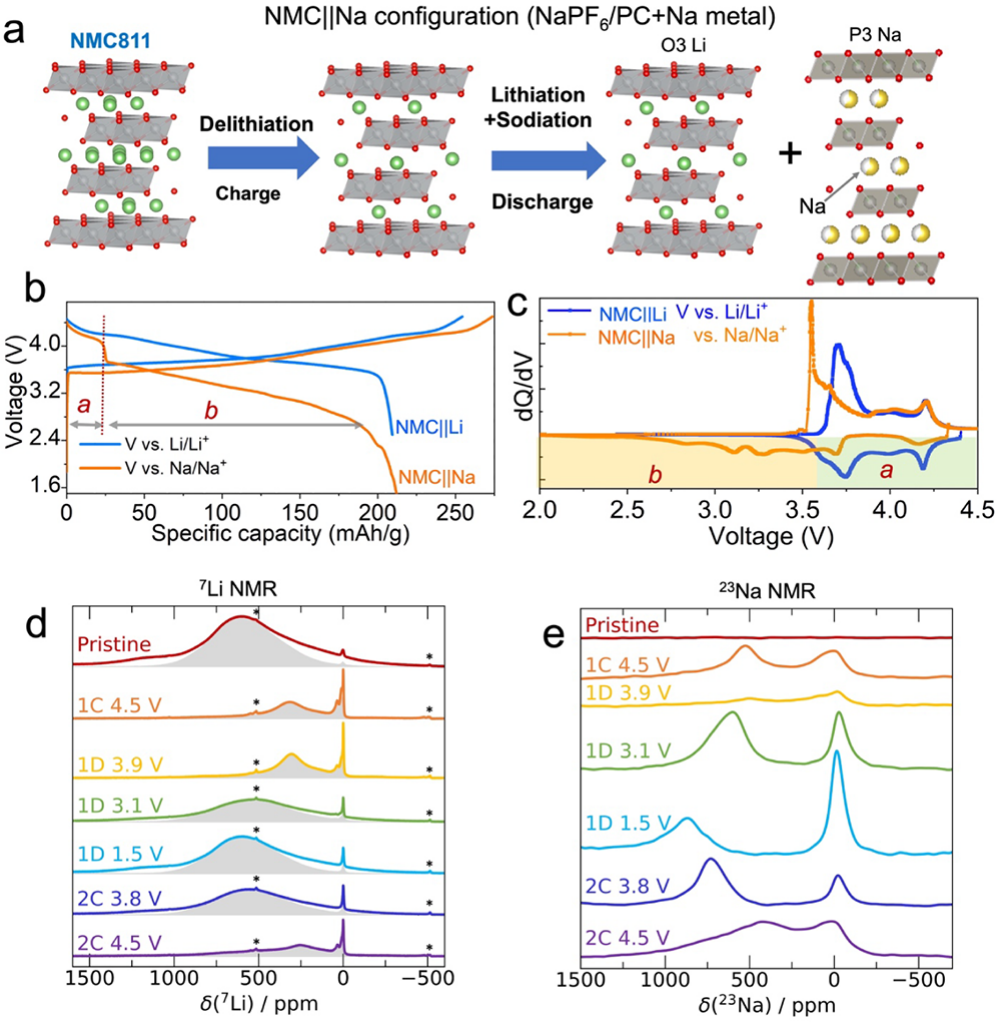
Electrochemical ion exchange in an NMC||Na cell (1 M NaPF_6_ in PC electrolyte and a Na metal anode). (a) Schematic of ion (de)intercalation from/into the NMC structure during the first charge–discharge cycle. First charge–discharge (b) voltage profiles and (c) differential capacity (*dQ*/*dV*) curves obtained on NMC||Na and NMC||Li (1 M LiPF_6_ in EC/EMC electrolyte and Li metal anode) cells at a current density of 20 mA/g. (d) ^7^Li and (e) ^23^Na spin echo NMR spectra collected at 60 kHz MAS and at 300 MHz (7.05 T) on pristine and cycled cathode samples extracted from NMC||Na cells. Spinning sidebands are indicated by an asterisk (*). ^7^Li pj-MATPASS isotropic spectra (with sidebands suppressed in this experiment) are shown as shaded regions in (d). The spectra are labeled according to the cycle number, and C and D represent charge and discharge, respectively. For example, 1C 4.5 V means stopped upon initial charge to 4.5 V, and 2C means stopped during the 2nd charge process. Each spectrum is scaled according to the number of moles of material in the rotor and the number of scans collected during the experiment.

We investigated the electrochemical exchange of Li ions with Na ions in a Ni-rich LiNi_0.8_Mn_0.1_Co_0.1_O_2_ (NMC hereafter) cathode material. In this study, we primarily focus on the one-cell configuration (NMC||Na), which uses a Na metal counter electrode and a Na-based electrolyte (Figures 1a and S1a), to understand the underlying ion exchange mechanism. We hypothesize that there is competing Li and Na ion (de)intercalation during extended cycling, which affects the phase evolution, redox chemistry, and ion transport properties of the mixed alkali metal layered structure.

When compared to a standard NMC Li-ion half-cell (NMC||Li), the NMC||Na cell displays a slightly lower potential (∼0.12 V) during the initial charge process (Figure 1b,c). The differences in the charge potentials between the two cells cannot be solely explained by the charging process at the cathode since Li is extracted from the NMC in both cases. Instead, they may be partially attributed to concurrent Li and Na plating at the Na anode during charging of the NMC||Na cell as opposed to only Li plating onto the Li anode in the NMC||Li cell. In fact, Na plating onto Na metal, occurs at about ∼0.3 V above Li plating onto Li metal, with a standard potential of −2.71 V vs SHE for Na/Na^+^ and −3.05 V vs SHE for Li/Li^+^. Upon initial discharge, the voltage profile of the NMC||Na cell exhibits a pronounced ∼0.5 V potential drop at ca. 20 mAh/g of capacity, dividing the discharge curve into two regions referred to as region *a*, spanning the 4.5–3.5 V potential range, and region *b*, spanning the 3.5–2.5 V potential range. This electrochemical behavior contrasts with that of a two-cell configuration (Figure S1c), whereby the NMC cathode is first charged using a Li metal counter electrode and a Li-containing electrolyte, the cell is then disassembled, and the recovered cathode is discharged against a Na metal counter electrode. Indeed, no voltage drop is observed on discharge of the two-cell configuration (Figure S1d), suggesting that the drop is associated with the intercalation of both Li and Na into the NMC structure. To identify the potential regions associated with the intercalation sequence of Li and Na ion into the NMC cathode, we examined voltage profiles obtained after a Li spiking test (addition of 0.1 M LiPF_6_ to the Na-based electrolyte used in the NMC||Na configuration in Figure S2). The results indicate that the capacity delivered over regions *a* (4.5–3.5 V) and *b* (2.5–3.5 V) in the discharge profile are sensitive to the relative concentrations of Li and Na ions in the electrolyte; specifically, Li ion intercalation dominates within region *a* while Na ion intercalation dominates within region *b*. The evolution of the (NMC||Na) cell voltage on subsequent cycles is plotted in Figure S3a. We find that the potential in the first half of the charge process (2.5–3.8 V) consistently decreases with cycling. While a continuous loss of capacity is observed in region *a* on discharge, a steady capacity increase and voltage decay are observed in region *b*. These findings agree well with the evolution of the cyclic voltammetry (CV) data (Figure S3b). We attribute the gradual evolution of the voltage profile upon extended cycling to the progressive exchange of Li with Na in the cathode structure. This is due to two reasons: (1) Li deposition on the Na anode should increase the cell voltage instead of decreasing it, and (2) Li ions extracted and diluted in the Na-based electrolyte can be minimally deposited on the Na anode. Thus, we do not anticipate the minor Li deposition on the Na anode to change significantly past the first cycle.

To confirm the continuous exchange of Li by Na in the NMC structure during extended cycling of the NMC||Na cell, we estimated the cation ratio in NMC cathode samples obtained during the first cycle and up to cycle 20 using scanning electron microscopy/energy dispersive spectroscopy (SEM-EDS) and inductively coupled plasma optical emission spectroscopy (ICP-OES). As shown in Figure S4, the Na atomic ratio in the cathode continuously grows over the first 20 cycles, at the expense of the Li atomic ratio. The evolution of the Na/Li ratio at various stages of cycling is consistent with the much larger reservoir of Na ions in the electrochemical cell, including the Na-based electrolyte and the Na metal anode, resulting in a continuous exchange of Li by Na over repeated charge/discharge cycles. Such a continuous electrochemical ion exchange process is further supported by the solid-state ^7^Li and ^23^Na nuclear magnetic resonance (NMR) results presented below.

^7^Li and ^23^Na spin echo solid-state NMR spectra (Figure 1d,e) of pristine and electrochemically delithiated NMC samples contain broad and highly shifted resonances, as well as much sharper resonances near 0 ppm. The former are attributed to Li/Na species intercalated into the bulk paramagnetic cathode, discussed below. The latter arise from secondary diamagnetic phases, e.g., Li/Na carbonate and/or (hydro)oxide phases remaining from synthesis or from electrolyte decomposition.^26−33^ The asterisks denote sidebands arising from fast rotation of the sample during NMR data acquisition, which are effectively suppressed in the ^7^Li pj-MATPASS NMR spectra^34^ overlaid with the solid ^7^Li spin–echo spectra in Figure 1d. The large chemical shifts of the ^7^Li and ^23^Na ssNMR signals are dominated by the paramagnetic (Fermi contact) shift proportional to the unpaired electron spin density delocalized from the 3*d* orbitals of nearby *TM* species (*TM* = Ni, Mn, and Co) onto Li/Na *s* orbitals via bridging O 2*p* orbitals.^26^ Each *TM*-O-Li/Na interaction gives rise to a unique shift contribution that depends on the TM oxidation state and interaction geometry, with the observed ^7^Li and ^23^Na shifts being the sum over all contributions from TM species in their first and second cation coordination shells in the rock salt structure. ^7^Li and ^23^Na NMR are thus highly sensitive to minute changes in the local environment of intercalating Li and Na ions. For example, the ^7^Li NMR signals observed for samples stopped on initial charge to 4.5 V (1C 4.5 V sample) and subsequent discharge to 3.9 V (1D 3.9 V) are lower in intensity and shifted upfield (toward lower ppm) compared to those observed in the pristine spectrum, indicating Li deintercalation and concurrent with TM oxidation.^35^ For the ^23^Na NMR spectra, while the pristine NMC cathode contains no Na, two broad and asymmetric ^23^Na signals near 430 and 11 ppm appear at 1C 4.5 V, suggestive of Na intercalation into the NMC interlayers. Furthermore, the presence of ^7^Li and ^23^Na signals at similar resonant frequencies in the 1C 4.5 V and 1D 3.9 V samples suggests a similar distribution of Li and Na environments at these two SOCs. Those surprising results can be reconciled if we consider spontaneous ion (mostly Na) uptake from the surrounding electrolyte into the highly charged and unstable NMC structure at 1C 4.5 V accompanied by metal reduction once the current is stopped for *ex situ* analysis and are not reflective of the processes occurring during continued battery operation.^36^ This interpretation is corroborated by the much weaker ^23^Na signal observed on subsequent discharge to 3.9 V. The 1D 3.9 V sample is more stable than the 1C 4.5 V sample and does not spontaneously intercalate ions from the surrounding electrolyte following cycling and thus provides a better picture of the actual amount of Na electrochemically inserted into the NMC structure at this SOC. We note that spontaneous ion uptake is validated by neutron diffraction pair distribution function (PDF), as shown in Figure S5. Li uptake into the NMC structure at the top of charge is likely minimal (considering the much larger Na reservoir in the electrolyte), and the increased ^7^Li ssNMR signal observed at 1D 3.9 V compared to 1C 4.5 V suggests that Li ions dominate the intercalation process at high voltage (>3.9 V), in good agreement with the preceding voltage profile analysis. We attribute the two Na resonances observed in the 1C 4.5 V charged and 1D 3.9 V discharged spectra to Na species in the interlayer space surrounded by different numbers of Mn, Ni, and Co species in their first and second cation coordination shells, or connected to these TM species via different *TM*-O-Li pathway geometries (as would be the case in the presence of octahedral and prismatic interlayer sites). On further discharge to 1D 3.1 V and to 1D 1.5 V, the broad ^7^Li and ^23^Na signals become more intense and shift downfield (toward higher ppm frequencies), indicating further coinsertion of Li and Na ions into the NMC structure accompanied by transition metal reduction. Notably, the 1D 1.5 V ^7^Li spectrum is nearly identical to that obtained on the pristine cathode (Figure S6a), but has a weaker signal intensity indicating that, while the distributions of Li environments in the pristine and 1D 3.1 V samples are similar, not all the Li initially present in the NMC cathode is reintercalated on discharge. These results further suggest that Na and Li ions occupy separate (inter)layers at low potentials; i.e., they do not mix. The cathode sample collected on subsequent state (2C 4.5 V) is once again unstable with respect to spontaneous ion uptake. Nevertheless, a comparison of the ^7^Li and ^23^Na spectra obtained on first (1C 4.5 V) and second (2C 4.5 V) charge (Figure S6b,c) suggests a lower Li content and a greater Na content in the cathode at 2C 4.5 V, consistent with the gradual replacement of Li by Na upon extended cycling. The Li content in the *ex situ* samples of interest was estimated from fits of the *ex situ* NMR spectra (see Figure S7), and the semiquantitative results indicate a gradual replacement of Li by Na during cycling, corroborating the SEM-EDS and ICP results discussed earlier.

### Structural Evolution

**Figure 2:**
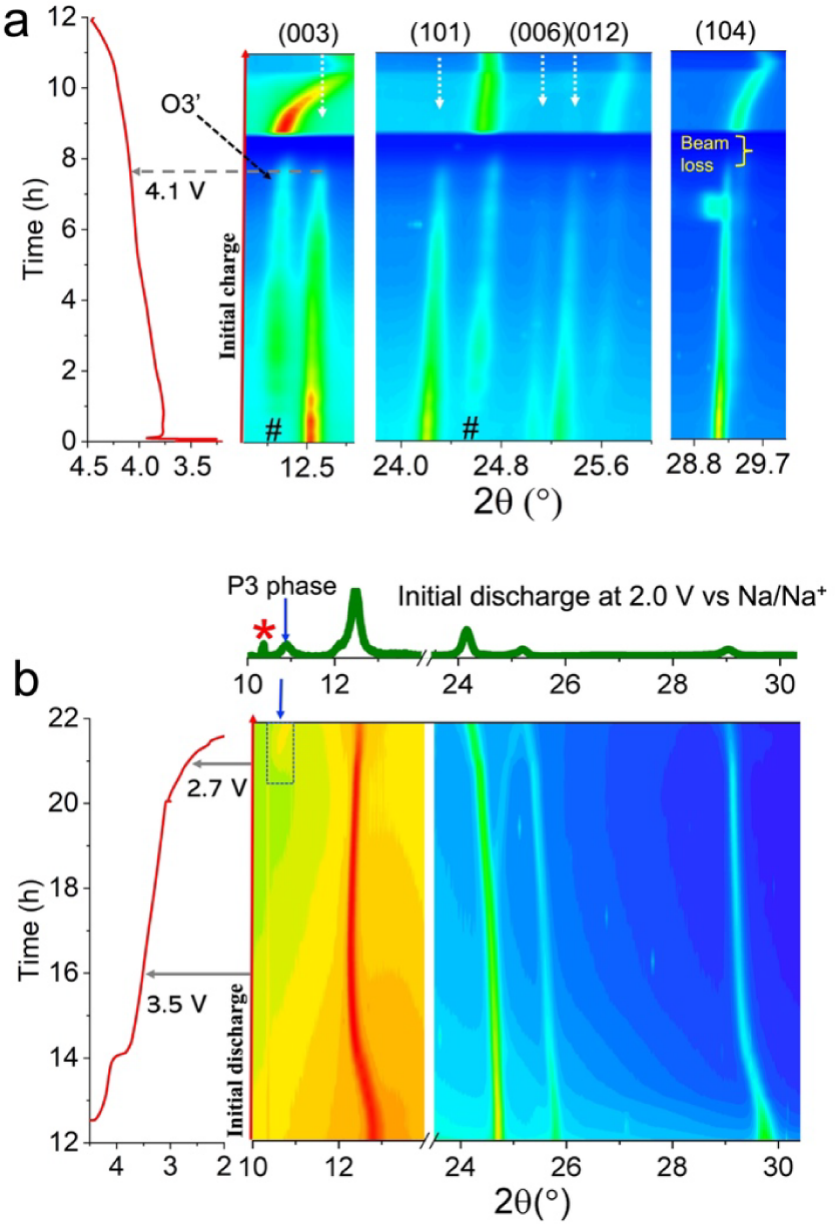
Structural evolution of NMC cycled in a NMC||Na cell. *Operando* XRD results were obtained during the initial charge (a) and discharge (b) processes. The cell was cycled at C/10 (20 mA/g) over the 1.5–4.5 V vs Na/Na^+^ range. The symbols “#” in (a) denote the emerging reflections of O’ phase, while “*” in (b) signifies the inherent peak of the *operando* cell since it persists consistently throughout the cycling process.

We performed *operando* synchrotron XRD to gain insight into the impact of competing Li and Na intercalation on the evolution of the long-range NMC structure in an NMC||Na cell (Figures 2 and S8). Li extraction from NMC in a Li half-cell (i.e., NMC||Li) leads to a continuous increase of the *c* lattice parameter^23,37−39^ as evidenced by a shift of the (00*l*) peaks toward lower angles and a shift of the (10*l*) peaks toward higher angles. In contrast, we observed a further splitting of the (003) and (101) peaks upon charging to 3.8 V, resulting in the emergence of two peaks at ∼12.3° and ∼24.6° attributed to a new O3′ phase (labeled with # in Figure 2a). The two (O3 and O3′) coexisting phases possess the same structure and symmetry but different *a* and *c* lattice parameters suggesting different Li contents (Figure S9). The phase separation observed here can be attributed to heterogeneous delithiation, as observed in prior studies.^35,40,41^ The O3′ phase steadily grows at the expense of the O3 phase on charge, as shown in Figure S9, and the O3 phase fully converts into O3′ by ∼4.1 V (Figures 2a and S8). Upon further charge to 4.5 V, the (003) peak of the O3′ phase shifts toward higher angles, suggesting *TM*–O layer glides and a phase transition from O3′ to O1. Despite the gradual slippage of the cell over the course of the experiment, which resulted in a loss of X-ray signal in the high voltage range, the cell still delivered a normal electrochemical voltage profile. Consequently, the slippage did not affect the overall structural evolution of the cathode, including the formation of a new P3–Na phase upon discharge.

On initial discharge of the NMC||Na cell (4.5–3.5 V), the (003) peak shifts back toward lower angles (Figure 2b), indicating an increase in the *c* lattice parameter (Figure S9) that is consistent with initial Li ion reinsertion into the structure and the O1 phase transforms back to the O3 phase (no intermediate O3′ phase is observed). Near 3.5 V, the (003) peak starts to shift toward higher angles (Figure 2b), indicating a contraction of the *c* lattice parameter. Upon further discharge to ∼2.7 V, a new peak appears at ∼10.6° (indicated by an arrow in Figure 2b), revealing the formation of a secondary layered phase. This secondary layered phase grows at the expense of the parent O3 phase on further discharge to 1.5 V. Overall, the initial rapid expansion of the structure along the *c* axis is concurrent with an O1 to O3 phase transition on Li insertion at high voltage and is followed by a more gradual contraction of the interlayers filled by further alkali metal intercalation, as is typically observed in single ion intercalating cathodes (Figure S9). Rietveld refinement of the *ex situ* XRD pattern obtained on the 1D 1.5 V sample (Figure S10) shows the coexistence of Li-based phase (Li_0.8_*TM*O_2_, 90 wt % of the sample) with an O3 structure, and of a Na-based phase (Na_0.5_*TM*O_2_, 10 wt % of the sample) with a P3 structure, hereafter referred to as O3–Li and P3–Na phases, respectively. The NMR results discussed earlier indeed showed that Li ion reintercalates into analogous Li sites as found in the pristine cathode, further supporting the conclusion that minimal to no Li is present in the P3–Na phase and that little to no Na is present in the O3–Li dominating phase. In addition, refinements of *ex situ* XRD patterns obtained at the end of the first and fifth cycles (Figure S10) confirm that Na steadily replaces Li on later cycles, with the P3–Na phase increasing to 20 wt % of the sample after the fifth cycle at the expense of the O3–Li phase.

### Phase Separation and Heterogeneity in the Redox Processes from Operando TXM

**Figure 3:**
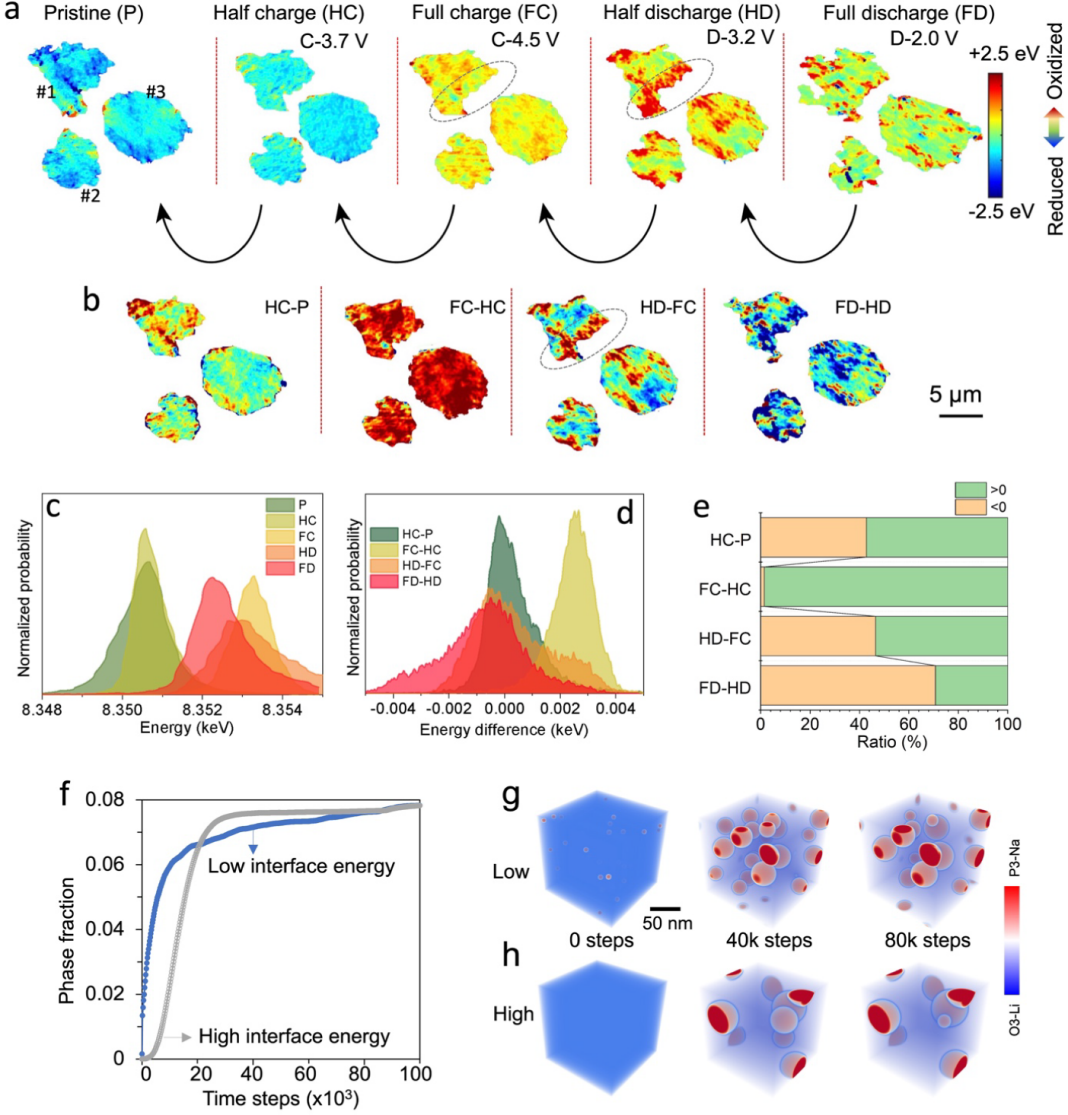
*Operando* TXM characterization of the evolution of Ni redox processes in NMC cathode particles (two-dimensional images) during the initial cycle in the NMC||Na cell, suggesting the presence of redox heterogeneity and phase separation. (a) NMC particles at five SOCs during the initial cycle at C/10 within a 2.0–4.5 V vs Na/Na^+^. Three secondary particles were randomly selected for the Ni redox analysis. The HC, FC, HD, and FD are collected at around 1C 3.7 V, 1C 4.5 V, 1D 3.2 V, and 1D 2.0 V, respectively. Each TXM measurement took ∼20 min. The energy scale refers to the relative energy change of the Ni K-edge white line. (b) Differential Ni oxidation state maps obtained between two SOCs, e.g., HC-P represents the difference between the half-charged (HC) and the pristine (P) states. (c) Distribution of Ni K-edge white line energies for the selected particles at various SOCs. (d) Distribution of Ni K-edge white line energy differences between two SOCs. (e) The areal fractions correspond to “reduced” and “oxidized” regions between two different SOCs. Here, a positive energy difference means that the white line difference of the Ni K-edge is above zero between two SOCs, indicating Ni oxidation in a local region, while a negative energy difference suggests Ni reduction. (f) Evolution of the volume fraction of the P3–Na phase when using two different P3–O3 interfacial energies (low and high) in phase-field simulations. Simulated morphologies of the P3–Na phase in the O3–Li matrix at different time stages in the case of low (g) and high P3–O3 interfacial energy (h).

Na intercalation and bulk diffusion during electrochemical cycling not only introduce complex structural changes, as discussed above, but also regulate the spatial distribution of redox processes within the NMC particles. Here, charge compensation mechanisms were first investigated using bulk sensitive *operando* hard XAS (Figure S11) and surface-sensitive *ex situ* soft XAS (Figure S12). Consistent with prior reports, the Ni redox couple is the main contributor to charge compensation processes in our NMC cathode, both in the bulk and at the surface of the cathode particles during the initial cycle, while Co and Mn are electrochemically inactive in the bulk but slightly reduced at the surface, a common phenomenon that involves surface oxygen loss and side reactions with the electrolyte.^39,42,43^ Thus, the subsequent X-ray absorption near-edge structure (XANES) experiments focus on Ni. By combining *operando* transmission X-ray microscopy (TXM) with XANES spectroscopic imaging (Figure 3), we can examine the distribution and propagation of Ni redox processes through the particles.^39,44,45^

The Ni oxidation state within three NMC secondary particles was continuously probed from the pristine state (P) to the following initial cycling in the NMC||Na cell (Figure 3a). Minor variations in the Ni oxidation states are detected within individual pristine NMC particles (Figures 3a,c and S13), likely resulting from the synthesis and/or from surface reduction by the organic electrolyte.^44−47^ Here, we discuss only the evolution of Ni oxidation states across the three particles at four representative SOCs, i.e., the half charged state at 3.7 V (HC), the fully charged state at 4.5 V (FC), the half discharged state at 3.2 V (HD), and the fully discharged state at 2.0 V (FD). The particles display a mosaic of domains with varying Ni oxidation states at all SOCs (Figure 3a), and each particle behaves differently (Figure S13). This variation was further quantified by the distribution of Ni absorption white line energies (the main absorption peak in XANES) observed at various SOCs (Figure 3c). The average oxidation state of Ni in the FD state is higher than that in the P state due to irreversible reactions (Figure 3c). The reduction of Ni in surface regions upon charging, which accounts for approximately ∼2% of the total 2D area probed in this measurement, can be attributed to the cathode–electrolyte interfacial reactions and localized oxygen loss (Figure 3a HC,e FC–HC).^48,49^ Additionally, during discharge, we find that certain regions of a particle are reduced, while others are counterintuitively oxidized. For example, regions that are yellow to red in the FC state change to red in the HD state (circled in Figure 3a,b), suggesting an increase in the Ni oxidation state upon discharge. As shown in Figure 3d, when comparing the HC state to the FC state (FC–HC), the oxidized regions account for over 98% of the total area, which aligns well with the expected oxidation of Ni on charge. On the other hand, upon discharge from the HD state to the FD state (FD–HD), the reduced regions only account for ∼70% of the total area, with the remaining ∼30% being oxidized instead (Figure 3e). Such observations contradict the expectation that TMs would undergo reduction upon discharging, which will be explained further explained later. It is worth noting that we followed the general procedure of controlling the radiation dose to minimize sample damage during the *operando* TXM experiment.^50^

Unsurprisingly, the heterogeneous Ni oxidation state distribution is accompanied by a nonuniform Na distribution at both the primary and secondary particle level, as indicated by elemental mapping in Figure S14. To provide insights into how the Na distribution may induce phase separation, we performed phase-field modeling to simulate the kinetics of nucleation and growth of the Na-dominating phase inside an NMC parent particle (Figure 3f–h). The specific interface energy (denoted as γ) critically determines the nucleation barrier (proportional to γ^3^ if assuming 3D homogeneous nucleation) and therefore the nucleation kinetics of the Na-dominating phase. As shown in Figure 3f, a low specific interfacial energy results in a faster nucleation and growth of the P3–Na phase, as well as the formation of finer-scale P3 phase within the parent phase (Figure 3 g,h). Such a two-phase morphology is qualitatively consistent with our TXM results (Figure 3b). Our phase-field simulations, together with the TXM results, therefore, suggest that the interface between the P3–Na phase and O3–Li phase has an intrinsically low energy, or equivalently, a high degree of structural coherency. Despite the low interfacial energy, the growth of the solid-state P3–Na phase within the O3–Li parent phase would nevertheless introduce internal stress hotspots and even cracks that may explain the observed reaction heterogeneity during discharge.

While heterogeneous redox processes have been observed in several studies, anomalous oxidation behavior during discharge has not been previously been reported. Herein, we hypothesize that the Ni redox evolution revealed by TXM is related to the nucleation and growth of the P3–Na phase within the initial ligand O3–Li phase. Specifically, Na intercalation induces a redistribution of Li at the local level, driven by the incompatibility of Li and Na in the same interlayer space, as revealed by our NMR analysis. Thus, there can be substantial variation in the Li and Na concentrations across different nanodomains, resulting in a wide distribution of Ni oxidation states. These nanodomains appear to undergo oxidation during Li/Na ion redistribution. Consequently, the intercalation of Na ions during discharge causes the formation of P3–Na nanodomains within the parent O3–Li phase. On further discharge, additional Na intercalation leads to the growth of the P3–Na domains that can be detected by XRD in the deep discharge state. We anticipate that changes in the local Li and Na concentrations occur throughout the discharge process. As mentioned earlier, the fully discharged cathode is composed of Li_0.8_*TM*O_2_ and Na_0.5_*TM*O_2_ phases as obtained from Rietveld refinements of the *operando* XRD results (Figure S10). In summary, upon discharge, phase separation results from a redistribution of Li and Na ions into separate domains, with a concurrent redistribution of Ni oxidation states to maintain local charge neutrality.^17,20,51^

### Ion Transport During Electrochemical Ion Exchange

**Figure 4:**
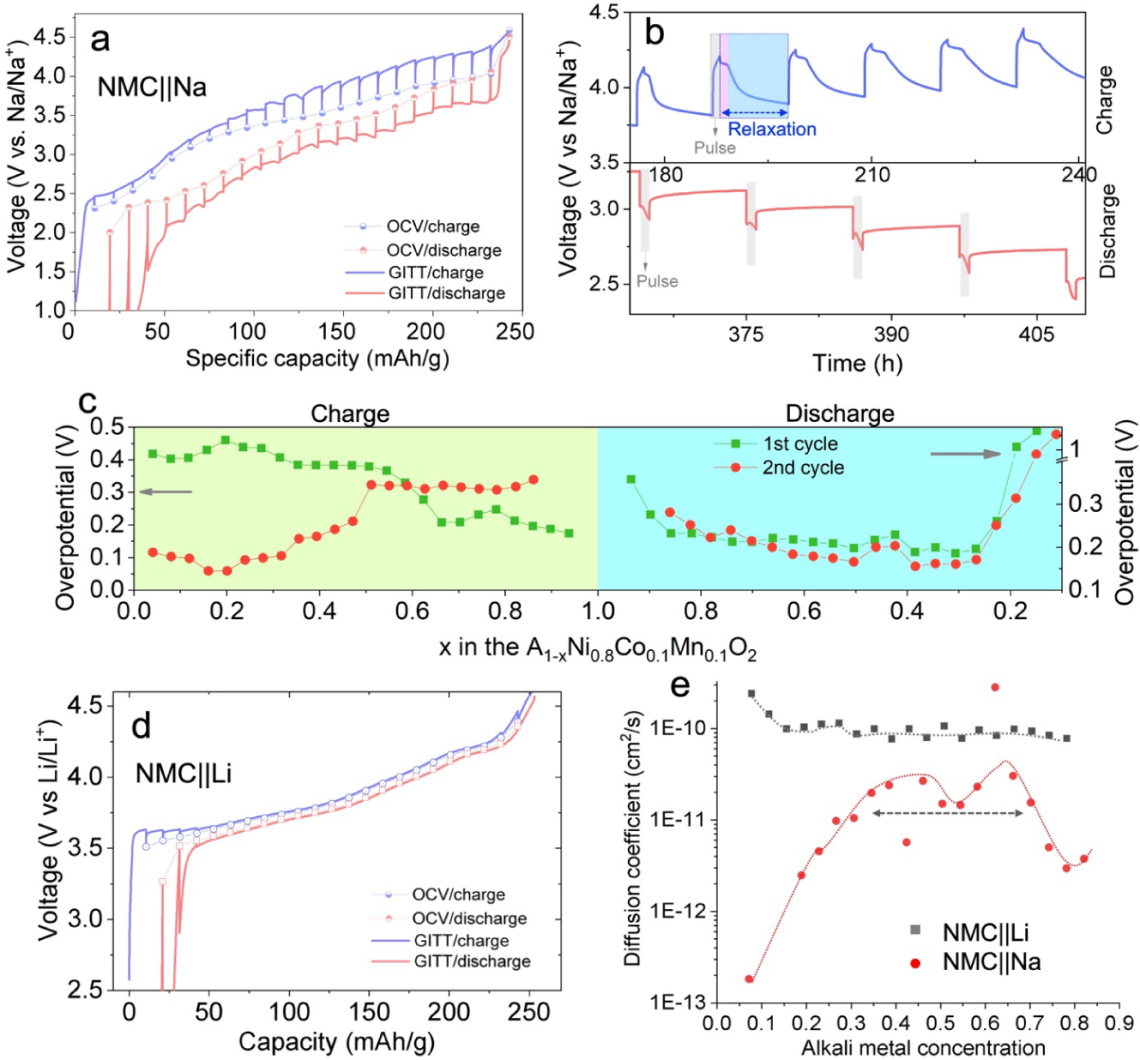
Analysis of the Li/Na ion transport properties in NMC||Na and NMC||Li cells. (a) GITT and pseudo-OCV curves of NMC cycled in an NMC||Na cell during the 2nd charge–discharge cycle. The cell was charged/discharged at C/20 for 1 h (pulse) and then rested for 10 h (relaxation). (b) Selected regions of the charge and discharge GITT profiles during the 2nd cycle, depicting voltage relaxation as a function of time. (c) Overpotential as a function of the concentration in alkali-ion (*A*), the left and right arrows point to the overpotentials during the charge and discharge, respectively. (d) GITT and pseudo-OCV curves were obtained on an (NMC||Li) cell. (e) Direct comparison of alkali-ion (*A*) apparent diffusion coefficient during discharge in the 2nd cycle in two different cells shown in (a) and (d). The two cells were cycled at C/20 (10 mA/g) for 1 h and allowed to rest for 10 h between each titration. Dashed lines are added to guide the visualization.

We employed the galvanostatic intermittent titration technique (GITT) to investigate the diffusion behavior of Li and Na ions during the electrochemical ion exchange process in the second cycle (Figure 4). The OCV (Figure 4a) curves obtained by joining the equilibrated potentials at different states of charge and discharge from the GITT measurement indicate significant hysteresis in the NMC||Na cell. Looking more closely at the relaxation processes, we find two relaxation processes in the voltage vs time profiles close to the top of charge (labeled in light purple and blue in Figure 4b top, respectively). We speculate that these processes may arise from the presence of two distinct time scales associated with the redistribution of Li and Na ion species within the bulk material to reach equilibrium at high voltages. Additionally, the discharge profile exhibits voltage fluctuations after each pulse (Figure 4b bottom), which we hypothesize are associated with the nucleation and growth of the P3–Na phase. The overpotential gradually decreases from 0.40 to 0.17 V during the initial charge but increases from 0.11 to 0.34 V during the second charge (Figure 4b,c). This suggests that Li/Na ion transport is sensitive to the chemical composition and structure, which changes continuously during electrochemical cycling as more Na replaces Li in the cathode. Upon discharge, the overpotential remains relatively constant around 0.19–0.28 V, until *x* < 0.25 where it significantly increases to approximately 1.65 V. This result indicates that further discharge/sodiation is kinetically hindered. For comparison, NMC was also cycled against a Li metal anode (NMC||Li) and a smaller gap between the charge and discharge voltage profiles was observed compared to the NMC||Na cell (Figure 4d), indicating a lower voltage hysteresis. In addition, we calculated the apparent diffusion coefficient (*D*) of the mobile ions (Figure 4e). In the NMC||Na cell, the values of *D* during discharge are within the range of 10^–12^–10^–11^ cm^2^/s, which is similar to many other reported layered cathode materials,^12,52^ but is 1 order of magnitude lower than that in the NMC||Li cell (Figure 4e). In summary, the presence of both Li and Na cations in the layered structure hinders charge–discharge kinetics, presumably due to sluggish phase boundary diffusion resulting from phase separation during cycling.

## Conclusion

Ion exchange dynamics, phase transformations, and redox processes were investigated upon electrochemical Li-to-Na ion exchange in the LiNi_0.8_Mn_0.1_Co_0.1_O_2_ (NMC) cathode. Using a combination of ^7^Li and ^23^Na solid-state NMR, *operando* X-ray diffraction, we found that the underlying mechanism is more complex than previously reported in the literature. Upon discharge of an electrochemically deionized NMC cathode in an NMC||Na cell, Li ion reintercalation occurs prior to Na ion intercalation, with Li and Na ions occupying separate interlayers. Furthermore, the discharge process involves phase separation, whereby a P3–Na phase nucleates and grows within the parent O3–Li phase. We employed *operando* TXM to examine the evolution of Ni redox processes in individual secondary particles and established spatial correlations between phase separation and Ni redox. We found that phase separation on discharge results from a redistribution of Li and Na ions into separate domains with a concurrent redistribution of Ni oxidation states to maintain local charge neutrality. The presence of small domains of the P3–Na phase within the parent O3–Li phase is consistent with a low interfacial energy, as suggested by the phase-field modeling results. The ion exchange process will likely be further influenced by the applied current density, particle size/morphology, and the transition metal compositions of the layered cathode.^53^ In summary, this study provides a comprehensive understanding of the complex processes underlying electrochemical cation exchange and not only contributes to the rational design of new intercalation materials but also provides a deeper understanding of alkali-ion metal separation through electrochemical ion (de)intercalation.

## Supporting Information Available

Experimental and modeling methods; electrochemical cycling data of battery cells in different configurations; EDS and ICP results; NMR characterization; synchrotron X-ray data analysis; STEM images (PDF)
