# Supplementary material for: Revealing the Chemical and Structural Complexity of Electrochemical Ion Exchange in Layered Oxide Materials
Source: J Am Chem Soc. 2024 Sep 17;146(39):26916–25. doi: 10.1021/jacs.4c08089 (PMC11457319; doi:10.1021/jacs.4c08089)
Supplement: Supplementary file 1 — ja4c08089_si_001.pdf [file ja4c08089_si_001.pdf]

Supporting Information for: Revealing the Chemical and Structural Complexity of Electrochemical Ion Exchange in Layered Oxide Materials

Linqin Mu,[a,b#*] Dong Hou,[a,c#] Emily E. Foley,[d#] Minyi Dai,[e] Jin Zhang,[f] Zhisen Jiang,[f] Muhammad Mominur Rahman,[a] Yanbao Fu,[g] Lu Ma,[h] Enyuan Hu,[i] Sami Sainio,[f] Dennis Nordlund,[f] Jue Liu,[j] Jia-Mian Hu,[e] Yijin Liu,[f] Raphaële J. Clément,[d*] Feng Lin[a,k*]

a) Department of Chemistry, Virginia Tech, Blacksburg, VA 24061, USA
b) School for Engineering of Matter, Transport and Energy, Arizona State University, Tempe, 85287
c) Institute for Materials Research and Innovation (IMRI), University of Louisiana at Lafayette, Lafayette, LA 70503, USA
d) Materials Department and Materials Research Laboratory, University of California Santa Barbara, CA 93106, USA
e) Department of Materials Science and Engineering, University of Wisconsin-Madison, Madison, WI 53706, USA
f) Stanford Synchrotron Radiation Lightsource, SLAC National Accelerator Laboratory, Menlo Park, CA 94025, USA
g) Energy Storage and Distributed Resources Division, Lawrence Berkeley National Laboratory, Berkeley, CA 94720, USA
h) National Synchrotron Light Source II, Brookhaven National Laboratory, Upton, NY 11973, USA
i) Chemistry Division, Brookhaven National Laboratory, Upton, NY 11973, USA
j) Neutron Scattering Division, Oak Ridge National Laboratory, Oak Ridge, Tennessee 37831, USA
k) Department of Materials Science and Engineering, Virginia Tech, Blacksburg, VA 24061, USA

Linqin Mu, Dong Hou, and Emily E. Foley contributed equally.
*Corresponding Authors
Emails: linqinmu@asu.edu; rclement@ucsb.edu; fenglin@vt.edu

Experimental information

Electrochemical testing

The cathode material of $LiNi_{0.8}Mn_{0.1}Co_{0.1}O_2$ (NMC, provided by Argonne National Laboratory) was processed into the electrodes as follows. The composite cathodes were prepared by spreading the slurry (N-methyl-2-pyrrolidone as the solvent) with active material (90 wt %), acetylene carbon (5 wt %), and PVdF binder (5 wt %), and cast on carbon-coated aluminum foils. The cathode electrodes were punched into disks of a diameter of 10 mm. The disks were then dried overnight at 120°C in a vacuum oven and transferred into an Ar-filled glove box. The cathode active mass loading was ~6 mg/cm^2. CR2032 coin cells were assembled in an Ar-filled glovebox using the composite cathode and a sodium or lithium foil anode, Whatman glass fiber (1827-047934-AH) as the separator, and 1 M $NaPF_6$ (Sigma-Aldrich, 98%) dissolved in propylene carbonate (PC) or 1 M $LiPF_6$ dissolved in ethylene carbonate (EC) and ethyl methyl carbonate (EMC) with the 2 wt % vinylene carbonates (VC) as the electrolyte (EC: EMC is 3:7). All the organic solvents were purchased from Gotion with water content under 1 ppm. All cells were cycled with an electrochemical workstation (Wuhan Land Company) at 22°C. 1C was defined as fully charging a cathode in 1 h, corresponding to a specific current density of 200 mA/g.

Two approaches were employed to obtain a layered structure with mixed Li and Na ions in the interlayer space electrochemically, either using a one-cell (NMC|Na) (**Fig. 1a**) or a two-cell (**Fig. S1**) configuration. In the one-cell configuration, a Na metal counter electrode and a Na-based electrolyte were used throughout the entire cycling. In the two-cell configuration, Li metal was used as the counter electrode during the first charge, along with a Li-based electrolyte. Then the cell was disassembled, and the charged cathode was transferred to a new cell with a Na counter electrode and a Na-based electrolyte for subsequent discharge and extended electrochemical cycling.

Characterization

The morphologies of the materials were investigated using a scanning electron microscope (SEM) (LEO FESEM) at an accelerating voltage of 5 kV. The Energy Dispersive X-ray Spectroscopy (EDS) and mapping were performed at an accelerating voltage of 10 kV. *Ex situ* XRD patterns for

the electrodes were collected at beamline 11-ID-C at APS with a wavelength of 0.1173 Å (105.7 keV). The patterns were recorded on a Perkin Elmer flat-panel amorphous-silicon 2D detector with a collection rate of 20 s. CeO_2 was used for calibration. The refinement of diffraction patterns was conducted using the software GSAS-II. Neutron PDF was performed on Nanoscale Ordered Materials Diffractometer (NOMAD) BL-1B, at the Spallation Neutron Source (SNS) at Oak Ridge National Laboratory. About 0.3 g of powder sample was loaded into a quartz capillary and measured for ~1 h. *Ex situ* soft XAS measurements were performed on the 31-pole wiggler beamline 10-1 at the Stanford Synchrotron Radiation Lightsource (SSRL). All spectra were normalized by the current from freshly evaporated gold on a fine grid positioned upstream of the main chamber. XAS samples were mounted on an aluminum sample holder with double-sided carbon tape in an Ar-filled glove box and transferred to the load-lock chamber in a double-contained container, using a glove bag purged with nitrogen for the transfer.

Operando XRD was carried out at Beamline 11-3 at SLAC at C/10 between 1.5-4.5 V vs. Na/Na^+. The wavelength was 0.97625 Å and LaB_6 was used for calibration. CR2032 coin cells with well-aligned Kapton windows were assembled in an Ar-filled glove box. The cathode electrode was made by mixing the NMC cathode powder (90 wt %), carbon black (5 wt %), and PTFE (5 wt %) to produce free-standing film electrodes with an active mass loading of ~6 mg/cm^2. Sodium metal was used as the anode and a 1 M $NaPF_6$ dissolved in propylene carbonate (PC) as the electrolyte. The same cell setup was used to perform *operando* hard XAS at Beamline 7-BM at NSLS-II with the operating current of C/5 within the 1.5-4.5 V vs. Na/Na^+. Pouch cells were assembled for the *operando* TXM experiment at Beamline 6-2 at SLAC. The cell was operated at C/10 within 2.0-4.5 V vs. Na/Na^+. Throughout the process of electrochemical ion exchange, we consistently gathered data on the morphology and XANES (X-ray Absorption Near Energy Structure) characteristics of the selected particles.

<u>Solid-state nuclear magnetic resonance (ssNMR) spectroscopy</u>

^{7}Li and ^{23}Na ssNMR spectra were collected on pristine and *ex situ* NMC powder samples using a Bruker Avance 300 MHz (7.05 T) wide-bore NMR spectrometer, at Larmor frequencies of 116.64 MHz and 79.39 MHz, respectively, and at room temperature. The data were obtained at 60 kHz magic angle spinning (MAS) using a 1.3 mm double-resonance HX probe. ^{7}Li and ^{23}Na NMR data

were referenced against 1 M aqueous lithium chloride (LiCl, δ(^{7}Li) = 0 ppm) and sodium chloride solutions (NaCl, δ(^{23}Na) = 0 ppm) and these samples were also used for pulse calibration. The data were processed using the Bruker TopSpin 4.0.8 software, and spectra were fitted using the DMfit software.[1] ^{7}Li spin echo spectra were acquired on all samples using a 90° radiofrequency (RF) pulse of 0.63 μs and a 180° RF pulse of 1.26 μs at 200 W. Recycle delays of either 0.05 s or 300 s were used to obtain spectra with very high signal-to-noise ratio or that were fully quantitative, respectively, on the NMC cathode samples of interest. ^{7}Li pj-MATPASS (projected Magic-Angle Turning Phase-Adjusted Sideband Separation)[2] isotropic spectra were also acquired on all samples using a 90° RF pulse of 0.63 μs at 200 W, with a recycle delay of 0.005 s. Transverse (T_2') relaxation times were obtained for all Li environments present in a sample from an exponential fit of the signal intensity decay upon increasing the delay time in an NMR spin echo pulse sequence. ^{23}Na spin-echo spectra were acquired on all samples using a 90° radiofrequency (RF) pulse of 0.37 μs and a 180° RF pulse of 0.74 μs at 200 W. A recycle delay between 0.005 ms and 0.5 s was used for the different NMC samples, with exact values optimized to ensure that the entire ^{23}Na signal was fully relaxed between pulses.

Phase-field modeling

Phase-field modeling is performed via the commercial software μ-Pro® to simulate the nucleation and growth of the Na-dominating phase inside an NMC parent particle. 3D spatial distribution of the local concentration of the Na-dominating phase c is used to describe the two-phase microstructure, where regimes with c=1 indicates the Na-dominating phase, c=0 indicates the NMC parent particle, and $0<c<1$ denote their interface. The total free energy F of the two-phase system is written as,

$$F = \int_0^v \left[f(c) + \frac{1}{2}\kappa_{ij}(\nabla_i c)(\nabla_j c) \right] dV \,. \tag{1}$$

Here $f(c)$ is the local chemical free energy density (J/m^3), which is the bulk thermodynamic driving force for the formation of the Na-dominating phase. As a first approximation, we adopt a relatively simple form of double-well potential for $f(c)$, given as,[3]

$$f(c) = c^2(1-c)^2. \tag{2}$$

This double-well potential yields a spinodal region of $0.21<c<0.79$. To simulate the kinetics of nucleation and growth of Na-dominating phase from the parent particle, the overall concentration

c_0 is set to be 0.1, which is outside the spinodal region. The second term in the integrand of Eq. (2) is the gradient energy density describing the short-range interaction between the diffusing species, where κ_{ij} is the gradient energy coefficient (J/m). The gradient energy density is part of the interface energy density. κ_{ij} is proportional to both the specific interface energy γ (J/m^2) and the interface width λ. In a 1D system, we have an analytical expression of κ=(3/2)$\gamma\lambda$. Since $f(c)$ is isotropic, the shape anisotropy of the equilibrium microstructure is determined by anisotropy of the specific interface energy (J/m^2). Here, we consider the growth of Na-dominating phase to be isotropic and therefore use an identical value for the diagonal components of the gradient energy coefficient matrix, *i.e.*, $\kappa_{xx} = \kappa_{yy} = \kappa_{zz} = \kappa$. In this work, κ = 0.5 and 1.0 are used for the simulation to study the influence of interface energy on the kinetics of nucleation and growth.

In our phase-field model, the formation of nuclei (in which c=1) in the NMC parent phase (in which c=0) is simulated based on the classical theory of nucleation and the growth where these nuclei are governed by Cahn-Hilliard type diffusion equations (with mass conservation). Assuming a homogeneous nucleation, the nucleation barrier $\Delta G^* = \frac{16\pi\gamma^3}{3(\Delta g)^2}$, where $\Delta g = f(c_0) + \left.\frac{\partial f(c)}{\partial c}\right|_{c=c_0}(1-c_0)$ is the thermodynamic driving force for the nucleation of the Na-dominating phase. Since the nucleation rate $j = N\exp\left(-\frac{\Delta G^*}{k_B T}\right)$, where N is the number of available nucleation sites, k_B is Boltzmann's constant, and T is the temperature, a lower specific interface energy will lead to a lower nucleation barrier and hence larger nucleation rate. In our model, we use Poisson's seeding to calculate the probability of nucleation (p) in each discretized grid at each time step, with $p = 1 - \exp(-j\Delta t)$, where Δt is the time interval (=0.1 herein). As more nuclei form, the number of available nucleation sites decreases, leading to a smaller j and hence a lower nucleation probability p. As a result, the kinetics will be dominated by the growth process which we simulate by numerically solving the Cahn-Hillard equation,

$$\frac{\partial c}{\partial t} = \nabla \cdot M_{ij}\nabla_j \frac{\delta F}{\delta c}; i,j = (x,y,z), \tag{3}$$

where M_{ij} is the mobility which should be a function of the mobilities of all the diffusing species as well as their local concentration.[3] For simplicity, we assume $M_{ij} = M(1 - ac^2)$ following refs.[4] where a=0 and a=1 correspond to bulk-diffusion-controlled and interface-diffusion-controlled dynamics, respectively. In the simulations, we set M=1 and a=0.9 to model interface-diffusion-

dominated growth dynamics. Equation (3) is numerically solved in a discretized 3D system of $128\Delta x \times 128\Delta y \times 128\Delta z$ using the semi-implicit Fourier spectral algorithm[5] under periodic boundary conditions. The grid sizes Δx=Δy=Δz=1 nm.

Figures

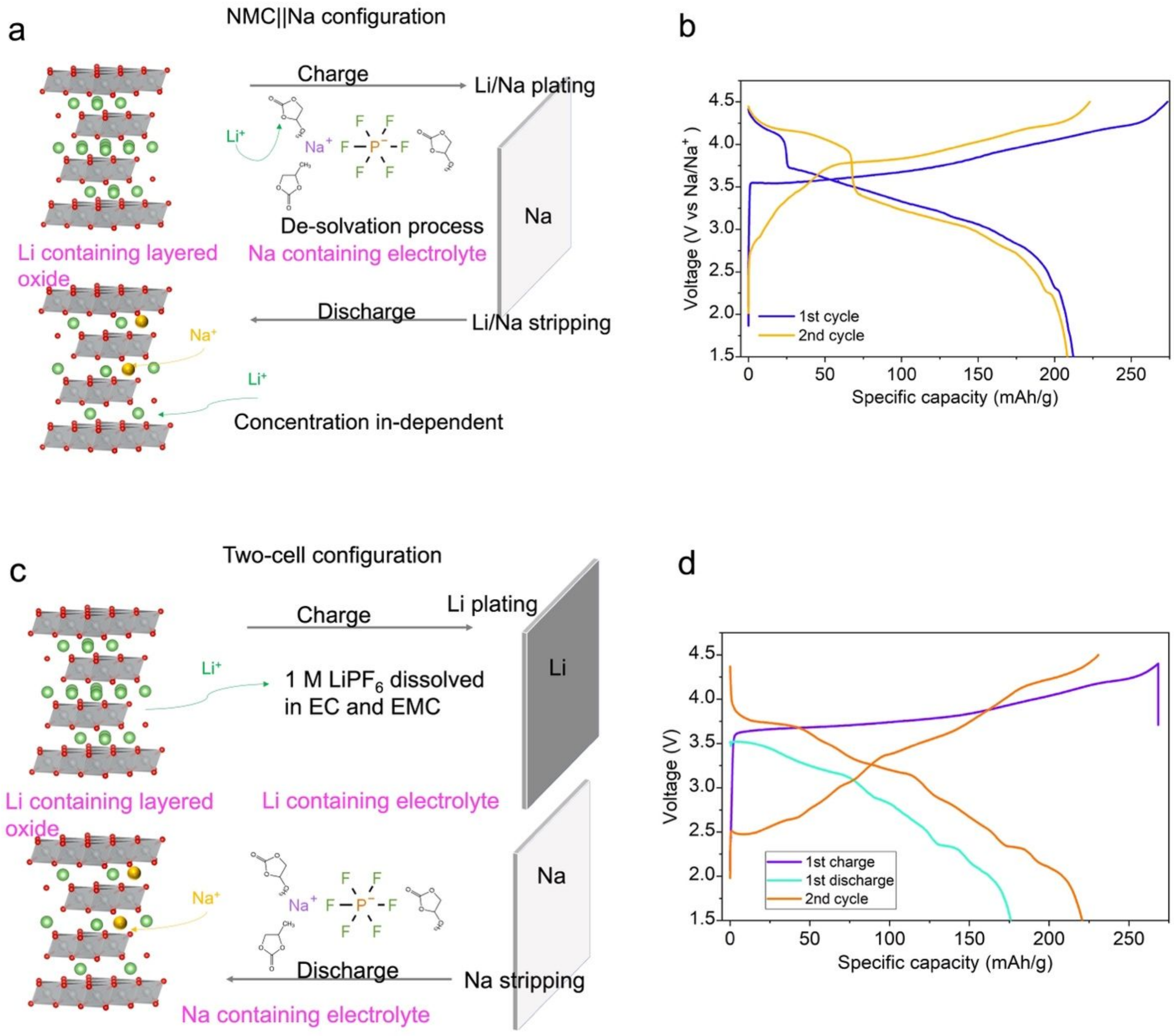

Figure S1 (a) One-cell NMC||Na configuration: electrochemical ion exchange takes place continuously in a cell consisting of NMC as the cathode, a Na metal anode, and 1M $NaPF_6$ dissolved in PC as the electrolyte. (b) Voltage profiles of the NMC||Na cell at C/10 (20 mA/g) over the 1.5–4.5 V range. (c) Two-cell configuration: the first cell was fabricated using NMC as the cathode, a Li metal anode, and 1M $LiPF_6$ dissolved in EC/EMC as the electrolyte (NMC||Li). The first cell was charged to 4.5 V vs. Li/Li^+ to electrochemically delithiated NMC. Subsequently, the first cell was opened, and the delithiated NMC was collected to be used as the cathode in the second cell. The second cell was assembled with the delithiated NMC as the cathode, Na metal

anode, and 1 M $NaPF_6$ dissolved in PC as the electrolyte. (d) Voltage profiles of the cell in the two-cell configuration at C/10 (20 mA/g) over the 1.5−4.5 V range. We switched the anode from Li to Na metal after the initial charge, thus, the voltage for the 1st charge is vs. Li/Li^+, while the voltage for the 1st discharge and 2nd cycle is vs. Na/Na^+.

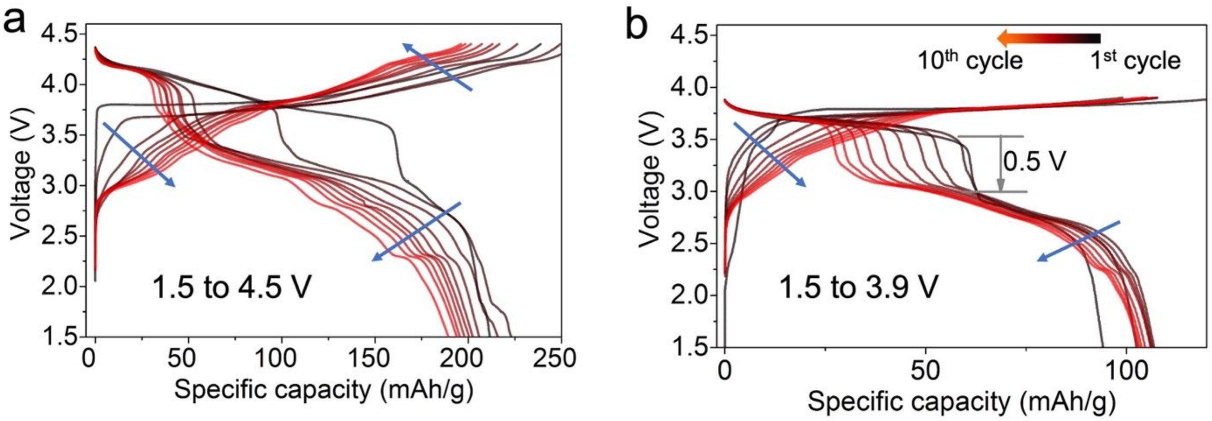

Figure S2 Charge/discharge profiles of NMC||Na cells, with the NMC as the cathode, 1 M $NaPF_6$ in PC as electrolyte, with the addition of 0.1 M $LiPF_6$ salt, and Na metal as the anode: (a) using a 1.5–4.5 V vs. Na/Na^+ voltage window, and (b) a 1.5–3.9 V vs. Na/Na^+ voltage window at a current rate of 20 mA/g. Arrows indicate the evolution of the voltage curves from the 1st to the 10th cycle. 0.1 M $LiPF_6$ salt was added to the Na-based electrolyte to test our hypothesis that Li ion intercalation into the NMC cathode mostly takes place at high voltage (>3.5 V), before the ~0.5 V drop in the discharge curve. If this hypothesis is correct, the high voltage capacity should be proportional to the concentration of Li ions in the electrolyte, at least in the first few cycles. The initial capacity from the high voltage range indeed significantly increases to more than 160 mAh/g, providing clear evidence for Li ion intercalation at > 3.5 V (Fig. S2a). Lowering the upper cutoff voltage to 3.9 V results in a similar initial increase in high voltage discharge capacity, accompanied by a 0.5 V voltage drop attributed to Na insertion, and a similar transition from Li-dominated to Na-dominated intercalation on extended cycling.

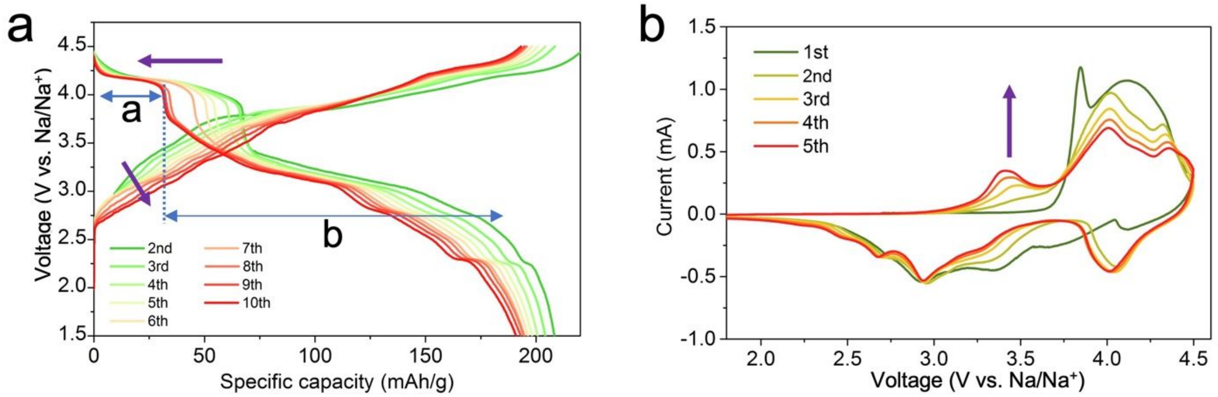

Figure S3 (a) Voltage profiles of the NMC||Na cell containing an NMC cathode, a Na metal anode, and a Na electrolyte at C/10 (20 mA/g) over the 1.5−4.5 V vs. Na/Na^+ potential window. (b) Cyclic voltammetry (CV) curves of the NMC||Na cell cycled at a scan rate of 0.1 mV/s. The arrows in (a) indicate the evolution of the voltage profile from the 2nd to the 10th cycle, indicating that the capacity decreases within region *a* (4.5−3.8 V) on discharge, while a continuous decrease in voltage within the 2.5−3.8 V range is observed upon cycling. The arrow in (b) indicates the emergence of an oxidation peak in the 3.4-3.5 V range upon extended cycling, which corresponds to the reduction peaks below 3.5 V observed on discharge. We anticipate that the redox processes in region *a* and *b* are primarily related to Li intercalation and Li/Na co-intercalation, respectively.

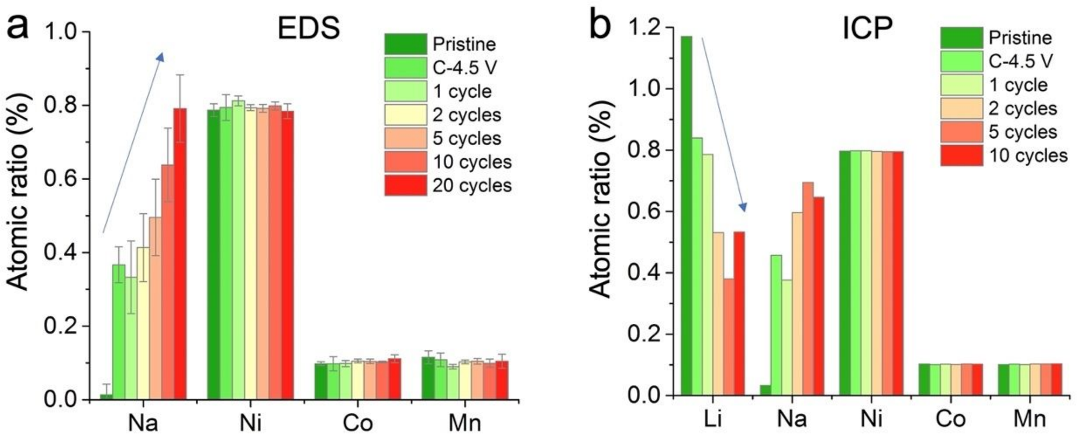

Figure S4 Atomic ratios of various elements in the NMC electrodes at various stages of cycling measured by (a) SEM-EDS, and (b) ICP-OES. The relative concentrations of Li and Na in the cathode obtained from these measurements confirm the continuous exchange of ions during extended cycling. The results also show that Na/Li exchange does not reach a plateau of 100% even after 20 cycles. It is worth noting that the Li atomic ratio in the pristine NMC was found to be over 1, which could potentially be attributed to the presence of surface Li_2CO_3. The Li concentration decreases throughout the cycling process. The presence of Na in the 1C-4.5 V sample (initial charge to 4.5 V) is attributed to spontaneous Na intercalation into the unstable, highly delithiated NMC phase during the relaxation process that occurs after the current is stopped. This phenomenon is discussed in more detail in the NMR results section in the main text.

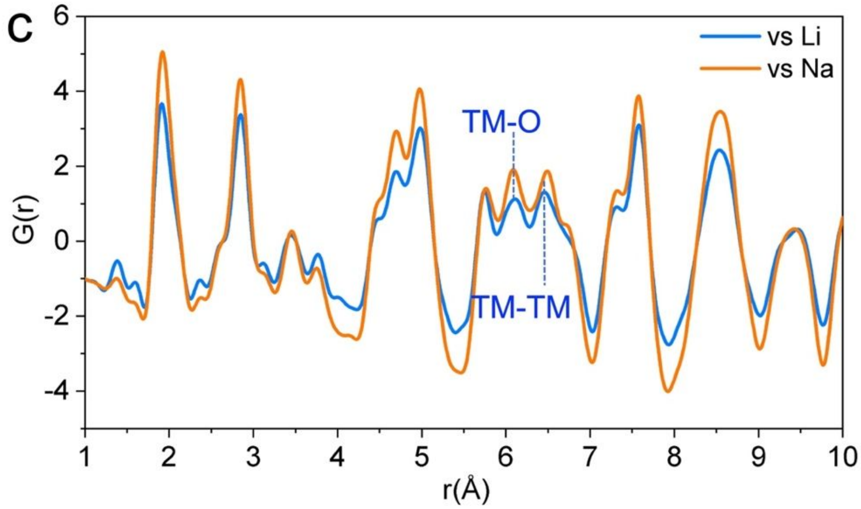

Figure S5 Experimental neutron PDF patterns collected on a 75% delithiated NMC sample after soaking in two different electrolytes for two weeks in an Ar-filled glove box. The Li electrolyte is 1 M $LiPF_6$ in EC/EMC, and the Na electrolyte is 1 M $NaPF_6$ in PC.

Ex situ neutron diffraction Pair Distribution Function (PDF) analysis was carried out to gain deeper insights into spontaneous ion uptake within the layered structure at high states of charge. NMC cathode samples were first charged to 4.5 V vs. Li/Li^+ to create Li vacancies (~75% delithiation), and then immersed in either Li or Na electrolyte for two weeks. Because Li and Na have a negative and positive scattering length, respectively, the increased intensity of the peaks located at ~2 Å and ~3 Å suggests that the nearest Li-O pair and Li-TM pairs are partially replaced by Na-O and Na-TM pairs when immersed in a Na electrolyte, indicating Na uptake into the structure during the relaxation process in a Na electrolyte. Moreover, a subtle shift of the peak at ~6.5 Å (interlayer TM-TM pairs) is observed, while the peak at ~6.1 Å, with a major contribution from intralayer TM-TM and TM-O distances, shows no change. Hence, we conclude that the initial incorporation of Na primarily alters the interlayer distance along the c-direction and exerts minimal influence on the atomic arrangements within the *ab* plane, primarily because of the limited quantity of Na involved. These discoveries complement our understanding of the interaction between Li/Na ions during the resting period in the surrounding electrolyte. However, it is worth noting that ND-PDF cannot distinguish the local distribution of Li/Na in the interlayer site.

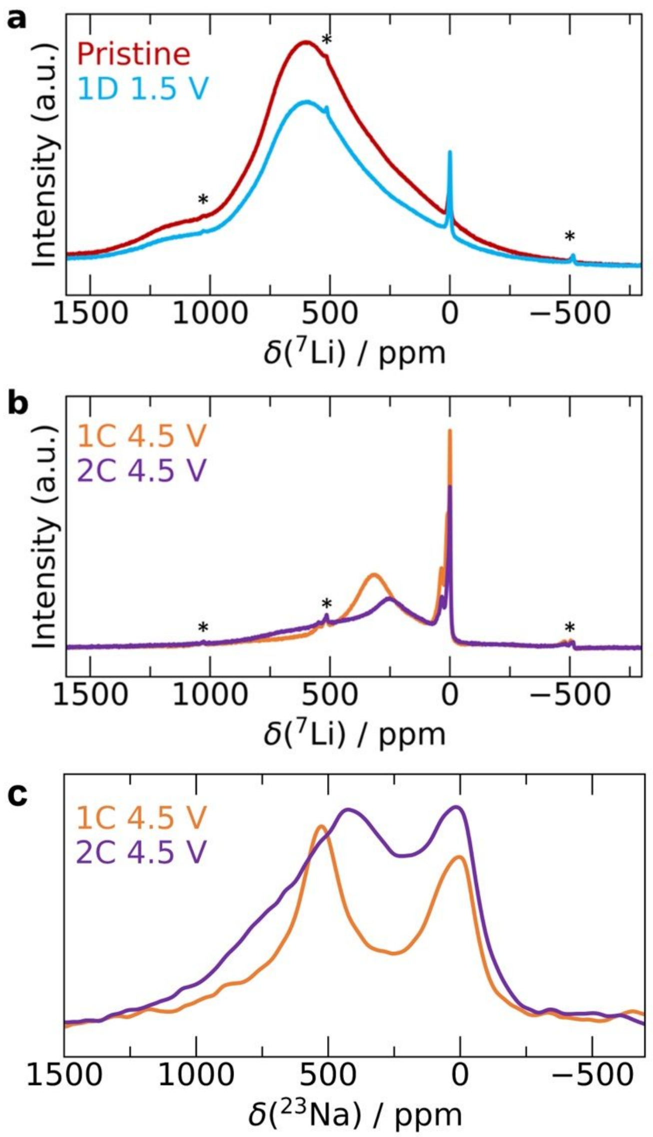

Figure S6. Comparison of solid-state NMR spectra reproduced from Fig. 1 and obtained on the: a) ^{7}Li spin echo NMR spectra of the pristine and 1D 1.5 V samples; b)) ^{7}Li spin echo NMR spectra of the 1C and 2C 4.5 V samples; c) ^{23}Na spin echo NMR spectra of the 1C and 2C 4.5 V samples. The spectra in a) and b) exhibit similar ^{7}Li NMR relaxation properties and can therefore be compared quantitatively. Each spectrum is scaled according to the number of moles of material in the rotor (using the molecular weight of $LiNi_{0.8}Mn_{0.1}Co_{0.1}O_2$ throughout) and the number of scans collected during the experiment. Spinning sidebands in the spin echo spectra are indicated by an asterisk (*).

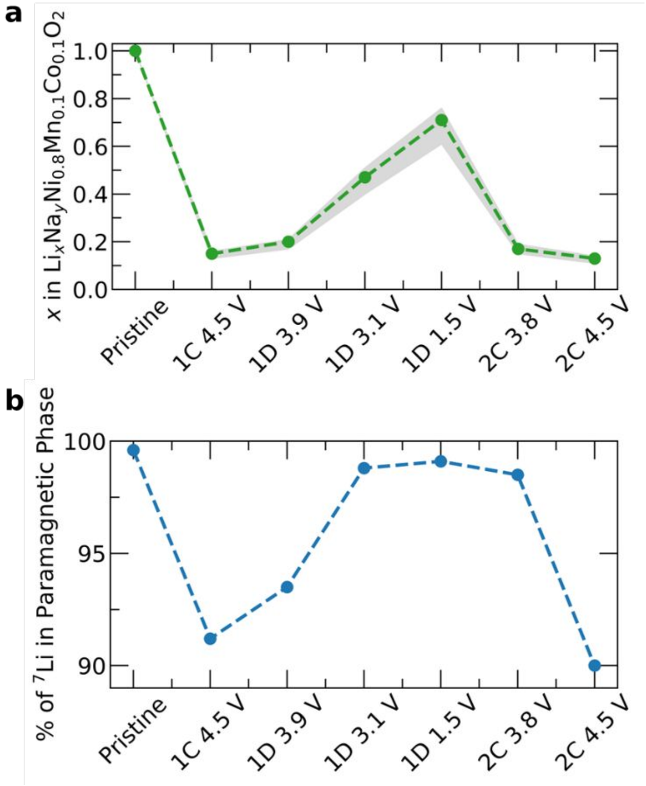

Figure S7 (a) Estimated Li contents in *ex situ* cycled NMC samples, obtained from analysis of the ^{7}Li solid-state NMR results shown in Fig. 1d. Quantitative ^{7}Li solid-state NMR spectra were fitted to extract the relative integrated intensity of the cathode signal (excluding sharp signals near 0 ppm associated with impurities and/or electrolyte degradation products) and then scaled to account for signal loss over the course of the NMR experiment due to transverse relaxation (T_2'). Results from these fits for Li present in paramagnetic phases are shown in (b) and are fully quantitative, indicating that more Li is present in diamagnetic phases at the top of charge, consistent with an overall decreased Li content and SEI formation at these high voltages. As signal loss when comparing across samples with very different relaxation properties is unable to be fully accounted for, as previously shown by Märker et al. for NMC811,[6] it is challenging to precisely quantify the Li content in the cathode phase. Specifically, all *ex situ* samples (besides 1D 1.5 V) exhibited significantly shorter T_2' values than the pristine sample resulting in loss of signal intensity that is unable to be accounted for when comparing integrated intensities across samples. Thus, the

estimated stoichiometry provided in (a) gives a lower bound for the Li content in the cathode material as signal is lost in the *ex situ* spectral integrated intensity due to significantly shorter T_2' values. These estimated stoichiometries were calculated based on the integrated intensity, amount of Li present in the cathode phase (b), and moles of ^{7}Li in the rotor – this step also inevitably introduced a error as the cathode molecular weight is dynamically changing upon cycling (the range in calculated stoichiometry based on varying molecular weights is shown as the grey shaded region, while the values computed using the molecular weight of $LiNi_{0.8}Mn_{0.1}Mn_{0.1}O_2$ is shown in green).

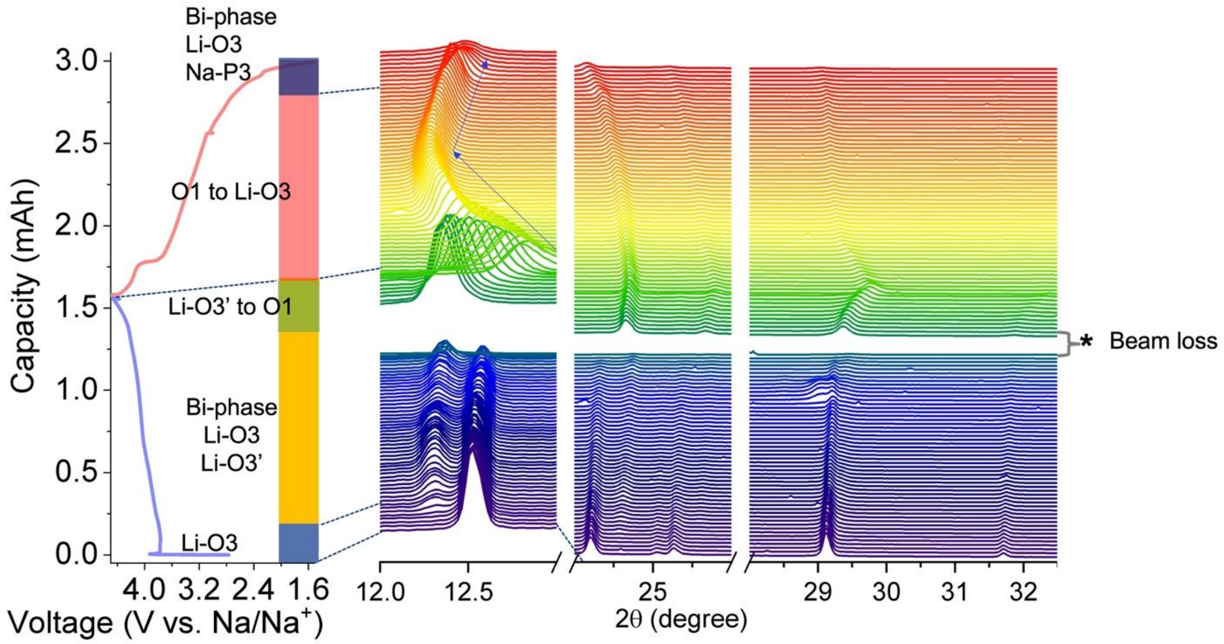

Figure S8 *Operando* synchrotron XRD was performed on the NMC||Na cell, which is consisted of NMC cathode, 1 M $NaPF_6$ in PC as the electrolyte, and Na as the anode. The coin cell was cycled at C/10 (20 mA/g) within 2.0–4.5 V vs. Na/Na^+. The left figure presents the voltage profile of the operando cell. The X-ray wavelength was 0.97625 Å. It is worth mentioning that there is a larger overpotential observed in the initial charging of the *operando* cell which may be attributed to a higher impedance caused by the Kapton sealed holes. As a result, the voltage profile does not

completely align with the one shown in Fig 1b. The new peak at ~10.6º corresponding to the P3-Na-dominating phase (shown in Fig 2b) is not included in this figure.

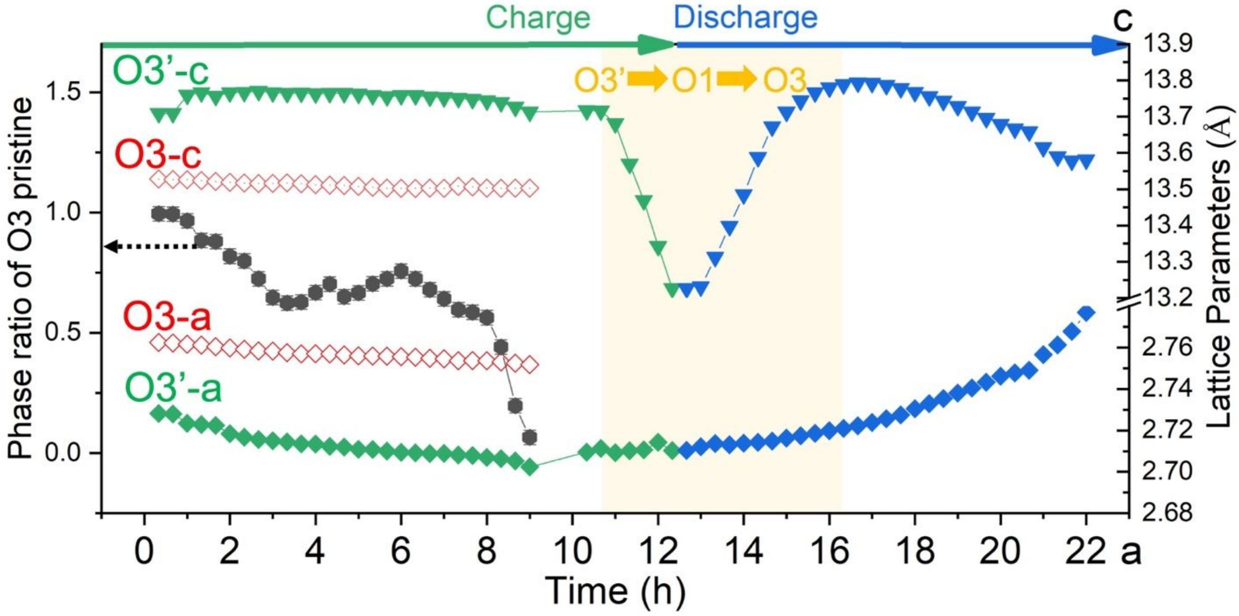

Figure S9 (a) Evolution of the *a* and *c* lattice parameters during the first cycle (y-axis on the right), phase fraction of the pristine O3 phase (dark grey dots, y-axis on the left). The red and green dots represent the pristine (O3) and distorted (O3') parent phase, respectively.

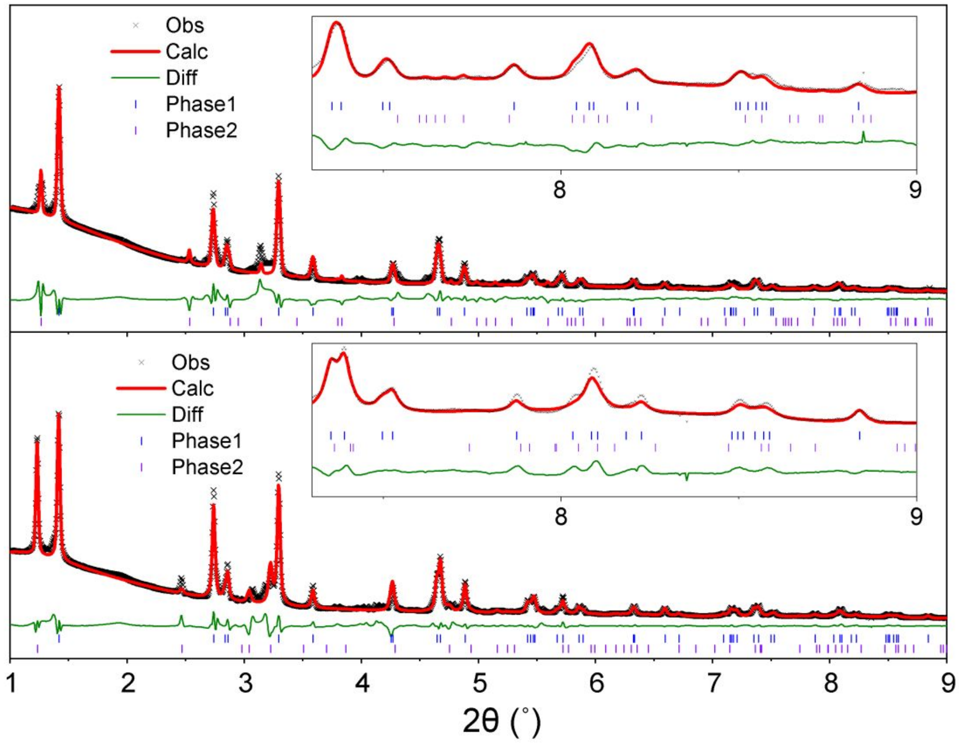

Figure S10 Rietveld refinement using a biphasic model (O3-Li phase + P3-Na phase) of NMC after 1 cycle (top) and 5 cycles (bottom) at the discharge state of 1.5 V vs. Na/Na^{+}. Both cathodes were cycled within an NMC||Na cell.

The refinement results are as follows:

(a) Weighted phase fraction: O3: 89.355 ± 0.84%, P3: 10.645 ± 0.38%, Goodness of fit: Rwp 6.48% and Rp 4.01%.

(b) Weighted phase fraction: O3: 79.794 ± 1.38%, P3: 20.251 ± 0.37%, Goodness of fit: Rwp 7.92% and Rp 4.98%.

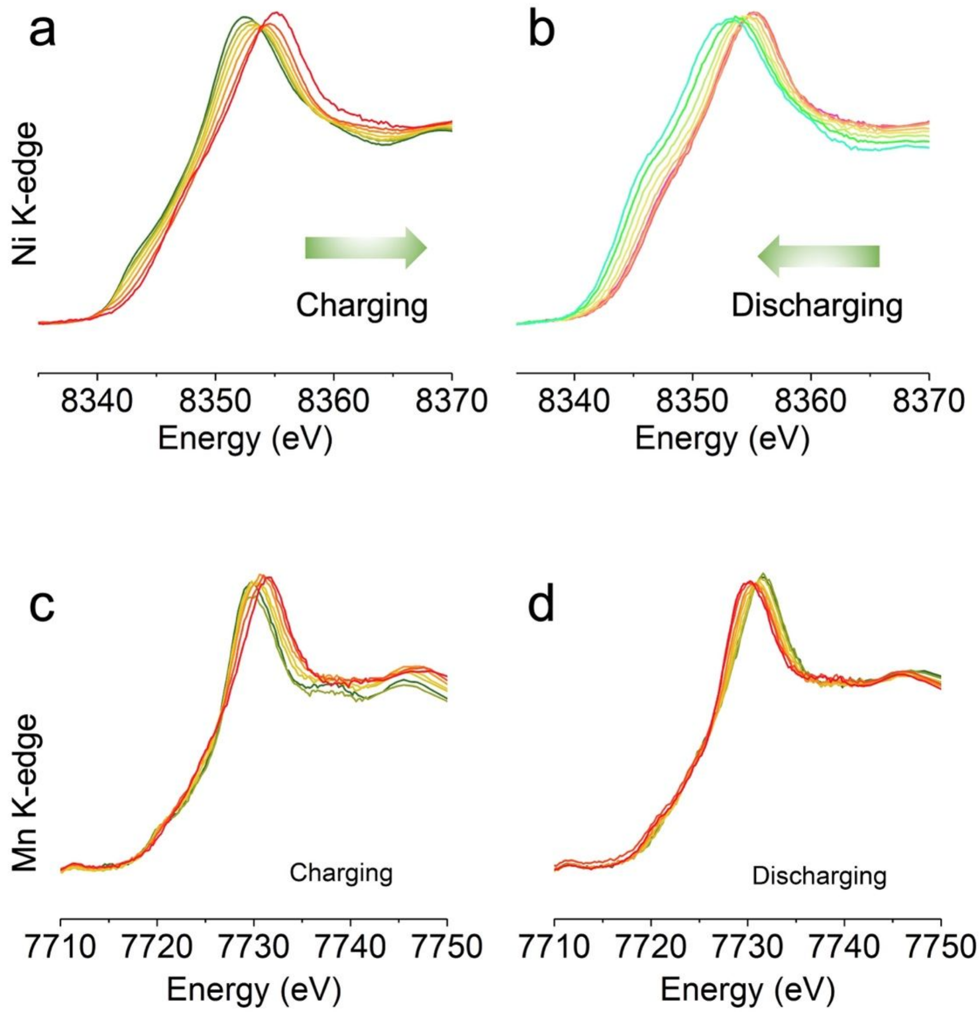

Figure S11 *Operando* hard XAS spectra of Ni (a, b) and Mn (c, d) K-edge of the NMC cathode in the NMC||Na cell within 1.5-4.5 V vs. Na/Na^+ at the C/5 (40 mA/g). The *operando* coin cell consisted of NMC as the cathode, 1 M $NaPF_6$ in PC as the electrolyte, and Na as the anode. Kapton tape was used to seal the window that can be penetrated by X-ray during the *operando* measurements.

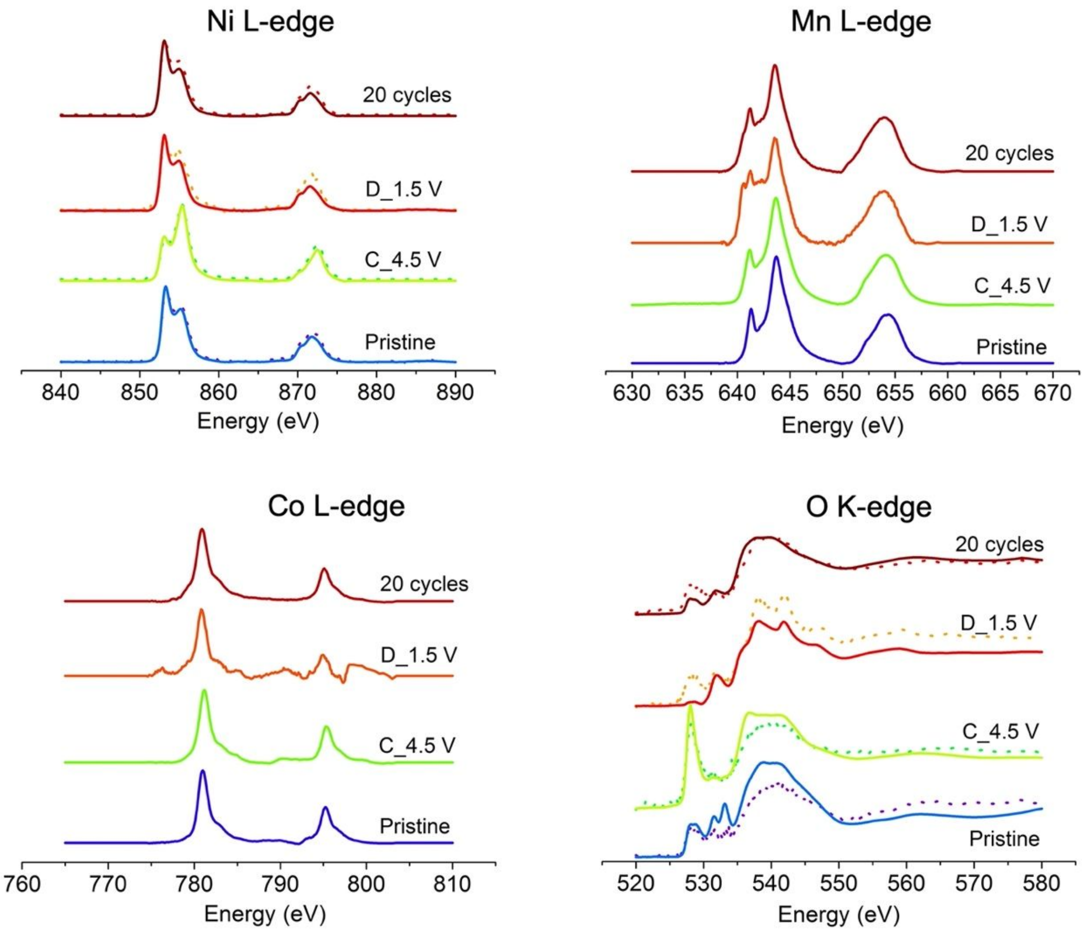

Figure S12 *Ex situ* Ni, Mn, and Co *L*-edge soft XAS spectra, O *K*-edge of the NMC cathode in the NMC||Na cell at various states. The NMC cathodes at different SOCs were cycled in the one-cell configuration with NMC as the cathode, 1 M $NaPF_6$ in PC as the electrolyte, and Na as the anode. Here we use solid and dash lines to represent the FY and TEY modes, respectively. For Mn and Co *L*-edge, only TEY mode was collected.

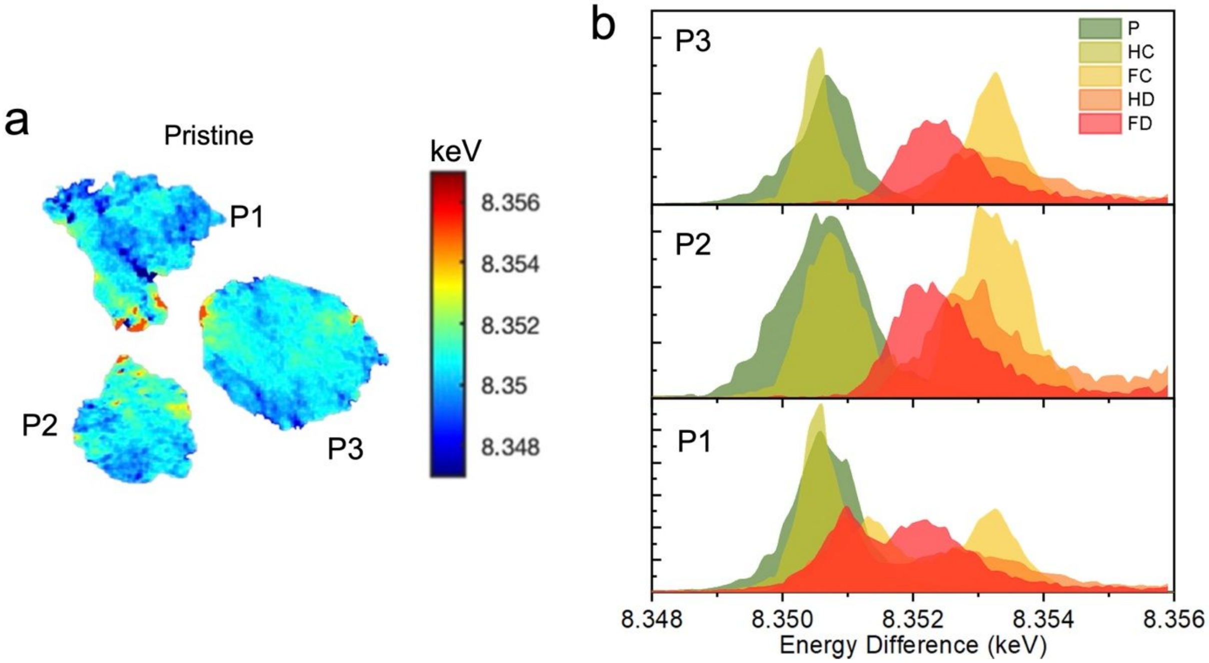

Figure S13 Charge heterogeneity probed using 2D TXM techniques on different secondary particles. (a) Three randomly selected adjacent particles in the pristine state, (b) Ni K-edge energy distribution profile for particles at four different SOCs. Ni oxidation state progressively increases and decreases during charging and discharging, as indicated by the overall Ni K-edge energy shift to high and low values, respectively. Interestingly, the Ni K-edge energy distribution profile split into two major peaks, which might be correlated with phase segregation where the Li-dominating and Na-dominating phases possess a relatively lower and higher Ni oxidation state, respectively.

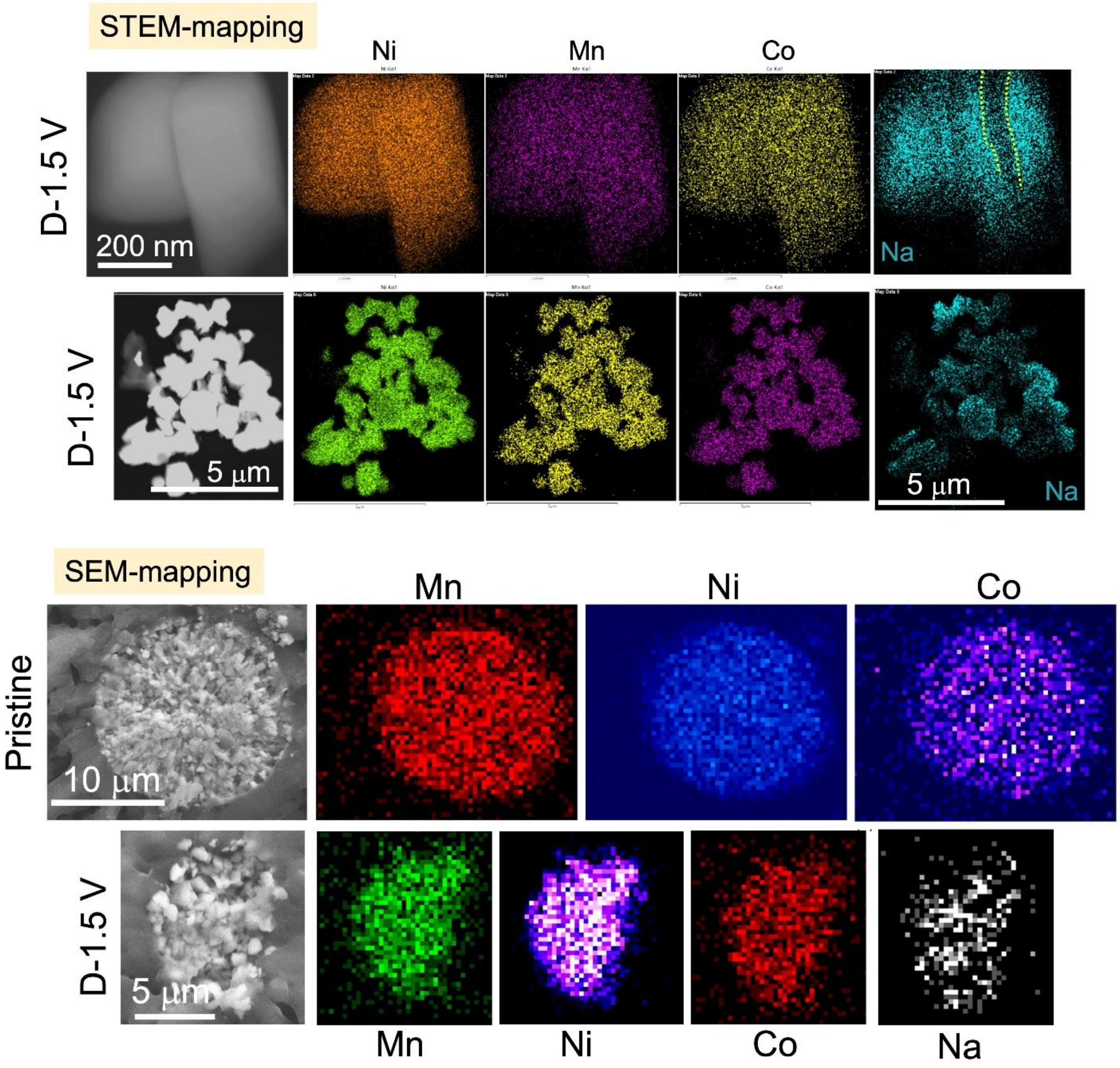

Figure S14 Mn, Ni, Co, Na elemental mapping. The NMC particles were cycled in the one-cell configuration, where NMC was the cathode, 1 M $NaPF_6$ in PC was the electrolyte and Na was the anode. D-1.5 V represents the discharge state in the first cycle.

References

(1) Massiot, D.; Fayon, F.; Capron, M.; King, I.; Le Calvé, S.; Alonso, B.; Durand, J.-O.; Bujoli, B.; Gan, Z.; Hoatson, G. Modelling one- and two-dimensional solid-state NMR spectra. *Magnetic Resonance in Chemistry* **2002**, *40* (1), 70-76. DOI: https://doi.org/10.1002/mrc.984.

(2) Hung, I.; Zhou, L.; Pourpoint, F.; Grey, C. P.; Gan, Z. Isotropic High Field NMR Spectra of Li-Ion Battery Materials with Anisotropy >1 MHz. *Journal of the American Chemical Society* **2012**, *134* (4), 1898-1901. DOI: 10.1021/ja209600m.

(3) Hu, J.-M.; Wang, B.; Ji, Y.; Yang, T.; Cheng, X.; Wang, Y.; Chen, L.-Q. Phase-Field Based Multiscale Modeling of Heterogeneous Solid Electrolytes: Applications to Nanoporous Li3PS4. *ACS Applied Materials & Interfaces* **2017**, *9* (38), 33341-33350. DOI: 10.1021/acsami.7b11292.

(4) Zhu, J.; Chen, L.-Q.; Shen, J.; Tikare, V. Coarsening kinetics from a variable-mobility Cahn-Hilliard equation: Application of a semi-implicit Fourier spectral method. *Physical Review E* **1999**, *60* (4), 3564-3572. DOI: 10.1103/PhysRevE.60.3564.

(5) Chen, L. Q.; Shen, J. Applications of semi-implicit Fourier-spectral method to phase field equations. *Computer Physics Communications* **1998**, *108* (2), 147-158. DOI: https://doi.org/10.1016/S0010-4655(97)00115-X.

(6) Märker, K.; Reeves, P. J.; Xu, C.; Griffith, K. J.; Grey, C. P. Evolution of Structure and Lithium Dynamics in LiNi0.8Mn0.1Co0.1O2 (NMC811) Cathodes during Electrochemical Cycling. *Chemistry of Materials* **2019**, *31* (7), 2545-2554. DOI: 10.1021/acs.chemmater.9b00140.
